# Genome-wide screening identifies new genes required for stress-induced phase 2 detoxification gene expression in animals

**DOI:** 10.1186/s12915-014-0064-6

**Published:** 2014-08-14

**Authors:** Helen M Crook-McMahon, Monika Oláhová, Emma L Button, Johnathan J Winter, Elizabeth A Veal

**Affiliations:** Institute for Cell and Molecular Biosciences, Newcastle University, Framlington Place, Newcastle upon Tyne, NE2 4HH UK

**Keywords:** Nrf2, p38, peroxiredoxin, arsenite, signal transduction, glutathione, aging

## Abstract

**Background:**

Phase 2 detoxification enzymes provide a vital defence against reactive oxygen species, including xenobiotic metabolites, which cause the oxidative damage involved in drug toxicity and many diseases. Hence, there is great interest in understanding how levels of these enzymes are regulated. CnC transcription factors, such as mammalian Nrf2, drive the expression of phase 2 enzymes and are activated as an important conserved response to oxidative stress and xenobiotics. For instance, the *Caenorhabditis elegans* Nrf2 orthologue, SKN-1, is activated in response to arsenite by the stress-activated p38-related kinase, PMK-1, leading to increased expression of phase 2 enzymes. Here we have used a genome-wide screening approach to identify other *C. elegans* genes that are required for stress-induced increases in phase 2 detoxification gene expression.

**Results:**

Taking advantage of the elevated phase 2 gene expression in a mutant lacking the peroxidase PRDX-2, we have identified many new genes that are required for stress-induced expression of *gcs-1*, a phase 2 enzyme critically required for glutathione synthesis. Significantly, these include genes previously implicated in resistance to ionizing radiation, longevity and responses to pathogenic infection. Many of these new candidate activators of *gcs-1* are also required for the stress-induced intestinal expression of other phase 2 genes. However, intriguingly, our data suggest other factors may be specifically required for the stress-induced expression of *gcs-1*. Notably, we demonstrate that the candidate activator TIR-1(SARM1) and the MAPKKK NSY-1(Ask1) are required for the arsenite-induced activation of PMK-1. However, our data suggest that the majority of candidates participate in novel mechanisms to promote gcs-1 expression. For example, the E4 ubiquitin ligase UFD-2(UBE4B) is dispensable for PMK-1 activation but important for maintaining nuclear levels of SKN-1, the stress-induced expression of multiple SKN-1-target genes and oxidative stress resistance.

**Conclusions:**

Here we present the first functional, genome-wide analysis identifying genes that are required for activation of phase 2 detoxification genes in an animal. Our study identifies potential new regulators of Nrf2, reveals that additional mechanisms promote the stress-induced expression of specific phase 2 detoxification genes and provides new insight into the relationships between these universally important stress defences, oxidative stress resistance and aging.

**Electronic supplementary material:**

The online version of this article (doi:10.1186/s12915-014-0064-6) contains supplementary material, which is available to authorized users.

## Background

Phase 2 conjugation reactions are vital for the biotransformation of lipophilic molecules to more soluble, excretable substances. By counteracting reactive metabolites of xenobiotics and oxygen (reactive oxygen species or ROS), phase 2 reactions provide important protection against the ROS-induced oxidative tissue damage that is associated with many diseases and aging. In addition, phase 2 reactions target diverse chemical substrates, thus presenting a significant barrier to both the activity and toxicity of therapeutic drugs [1]. Phase 2 reactions are also involved in the biosynthesis of hormones and inflammatory mediators [2]. Enzymes supporting phase 2 detoxification reactions, such as those involved in the biosynthesis of glutathione (e.g., γ-glutamylcysteine synthetase or GCS) and its conjugation to substrates (e.g., glutathione *S*-transferases or GSTs), are rapidly induced following exposure to stressful stimuli. This response is highly conserved, reflecting the key role of phase 2 detoxification reactions in protecting cells against ROS-induced damage. Indeed, there is growing evidence that increased levels of phase 2 enzymes have beneficial effects by preventing adverse drug reactions, protecting against carcinogens and promoting longevity [1,3,4]. On the other hand, increased phase 2 defences can also have negative consequences, for example, rendering tumour cells resistant to treatment [5,6].

Given the medical significance of phase 2 reactions, there is substantial interest in elucidating how these important stress and drug defences are regulated. Studies of the mechanisms regulating inducible phase 2 gene expression have focused upon the regulation of the Cap and Collar (CnC) transcription factor family that is important for this induction [7,8]. CnC transcription factors, such as Nrf2 (nuclear factor (erythroid-derived 2)-like 2) in mammals and SKN-1 in *Caenorhabditis elegans*, mediate transcriptional control of phase 2 genes. The activity of these transcription factors is regulated at multiple levels, including transcription, translation, nuclear localization and protein stability [7]. For example, the activation of Nrf2 in response to certain xenobiotics is mediated by chemical modifications of Keap1 that prevent it from targeting Nrf2 for degradation [5,7,9]. However, the mechanisms by which other xenobiotics and endogenous reactive chemicals induce the up-regulation of phase 2 genes are poorly understood.

Studies of *C. elegans* have provided important insight into the regulation of phase 2 genes*.* SKN-1, the *C. elegans* orthologue of Nrf2, activates expression of phase 2 genes, including γ-glutamylcysteine synthetase (*gcs-1*), which catalyses the rate-limiting step of glutathione synthesis [10]. In *C. elegans*, the intestine is the first line of defence against xenobiotics. SKN-1 in the intestine is activated in response to stress (e.g. arsenite) to increase intestinal expression of *gcs-1* and other phase 2 detoxification genes. Several signalling pathways regulate levels of active SKN-1. For example, phosphorylation of SKN-1 by the stress-activated mitogen-activated protein kinase (MAPK), PMK-1, is important for stress-induced increases in nuclear SKN-1 that result in increased phase 2 gene expression and oxidative stress resistance [11]. In contrast, insulin signalling and GSK-3-mediated phosphorylation both inhibit SKN-1 [4,12], as does interaction with the WD40-repeat protein WDR-23 [13]. However, the identification of genes that influence *gcs-1* expression by SKN-1-independent mechanisms [14] suggests the existence of additional mechanisms for regulating phase 2 detoxification gene expression.

We have previously found that in the absence of the 2-Cys peroxiredoxin, PRDX-2, which detoxifies peroxides, expression of phase 2 detoxification genes (e.g., *gcs-1*) and resistance to arsenite-induced oxidative stress are increased. For example, a stress-inducible *gcs-1p::gfp* transcriptional reporter is expressed at significant levels in the intestine of *prdx-2* mutant *C. elegans* even under normal growth conditions. Notably, this increased *gcs-1* expression and arsenite resistance are only partially dependent on SKN-1 [14]. Hence, to identify new regulators of stress-induced phase 2 gene expression, we have conducted a genome-wide RNA interference (RNAi) screen to identify genes required for the elevated intestinal *gcs-1p::gfp* expression in a *prdx-2* mutant. As predicted, a significant number of these genes are also required for the increased intestinal expression of *gcs-1p::gfp* in wild-type animals under stress conditions (arsenite exposure). Partial characterization of the most robust candidates reveals that our screen has identified new activators of PMK-1 and SKN-1, which are important for the arsenite-induced expression of multiple phase 2 genes and arsenite resistance. However, our data suggest that the majority of candidate genes may promote arsenite-induced *gcs-1* expression by alternative mechanisms, outside or downstream of the PMK-1/SKN-1 pathway. Indeed our study reveals unanticipated, additional complexity in the regulation of phase 2 detoxification systems and stress resistance in animals.

## Results

### Genome-wide RNAi screening identifies genes regulating *gcs-1p::gfp* expression

**Figure 1:**
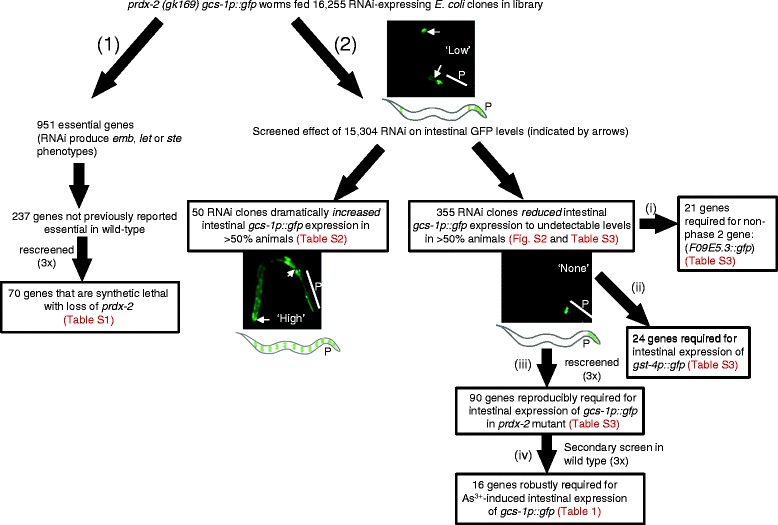
**Results of RNAi screening** **.** RNAi screening identified genes that are (1) synthetic lethal with *prdx-2* or (2) required for stress-induced, intestinal expression of a phase 2 detoxification transcriptional reporter gene (*gcs-1p::gfp*). First, 16,255 RNAi clones were screened in *prdx-2 gcs-1p::gfp* background, which contains increased, detectable ‘low’ levels of intestinal GFP (indicated by arrows for representative animals and illustration) in addition to wild-type levels of constitutive pharyngeal GFP expression (indicated with ‘P’) [14]. Of the 951 RNAi clones that produced embryonic lethal (*emb*), lethal (*let*) or sterile (*ste*) phenotypes (and hence too few *prdx-2* mutant progeny for *gcs-1p::gfp* to be scored), 237 had not previously been reported to affect growth or reproduction in screens of wild-type animals [15]. Following re-screening of these 237 genes three times, 70 RNAi clones repeatedly produced *emb*, *let* and/or *ste* phenotypes (Additional file 1: Table S1). Of the 15,304 wells containing at least 40 viable progeny in the initial screen, 50 RNAi clones were scored that increased intestinal *gcs-1p::gfp* expression, such that more than 50% of animals in the well had a ‘high’ level of *gcs-1p::gfp* expression throughout the intestine (see representative animal and illustration) (Additional file 1: Table S2). Another 355 RNAi clones reduced *gcs-1p::gfp* expression, such that more than 50% of animals in these wells had no detectable intestinal *gcs-1p::gfp* expression (‘None’; see representative animal and illustration) (Additional file 1: Table S3). These 355 RNAi clones were re-screened three times and subjected to secondary screens: (i) 21 clones were discovered to reduce the intestinal expression of non-phase 2 gene *F09E5.3::gfp* [16]; (ii) 24 clones reduced intestinal *gst-4p::gfp* expression (Additional file 1: Table S3); (iii) 90 clones reduced *gcs-1p::gfp* expression in *prdx-2* mutants on each of four separate occasions (Additional file 1: Table S3); and (iv) 16 of these clones reduced arsenite-induced intestinal *gcs-1p::gfp* expression in wild-type worms (N2) in each of three trials but did not reduce *F09E5.3::gfp* expression (Table 1). GFP, green fluorescent protein; RNAi, RNA interference.

To identify new activators of phase 2 gene expression, particularly genes that are required for the stress-induced intestinal expression of these genes, we performed a genome-wide RNAi screen for genes required for the elevated intestinal expression of *gcs-1p::gfp* in the *prdx-2* mutant background [14]. As intestinal expression of *gcs-1p::gfp* was assessed in the progeny of RNAi-treated worms (Figure 1), this prevented us from examining whether RNAi targeting of essential genes affected *gcs-1p::gfp* expression. However, incidentally, this allowed the identification of 70 genes that may be specifically required for development or reproduction in the absence of *prdx-2* (see Additional file 1: Table S1 and Additional file 2: Figure S1).

Although the purpose of this screen was to identify genes that were required for the elevated intestinal expression of *gcs-1p::gfp* in *prdx-2* mutant animals, in the course of screening, 50 RNAi clones were noted that increased the expression of *gcs-1p::gfp* still further (see Additional file 1: Table S2). These included RNAi targeting genes encoding E01A2.1, a glutamate-cysteine ligase regulatory subunit and WDR-23, a WD40-repeat protein that negatively regulates SKN-1 [13]. Both of these genes were identified by previous screens for inhibitors of phase 2 detoxification gene expression, confirming that our screening conditions were effective at identifying regulators of *gcs-1p::gfp* [13,17]. RNAi targeting glutathione synthetase (*M176.2*) also increased *gcs-1p::gfp* expression, providing further evidence that feedback control acts to increase GCS-1 levels when glutathione synthesis is inhibited [17].

### Genes required for *gcs-1p::gfp* expression overlap with genes required for resistance to ionizing radiation and longevity

It was found that 355 RNAi clones (approximately 2.3% of those screened) prevented intestinal *gcs-1p::gfp* expression (Figure 1 and see Additional file 1: Table S3). Secondary screens determined that 21 of these genes were also required for the intestinal expression of a transcriptional GFP reporter of a non-phase 2 gene, indicating a broader role in intestinal gene expression (see Additional file 1: Table S3). Accordingly, these genes were eliminated as candidate phase 2 gene regulators. Importantly, this analysis indicated that the majority of targeted genes are not generally required for intestinal gene expression or GFP stability.

**Figure 2:**
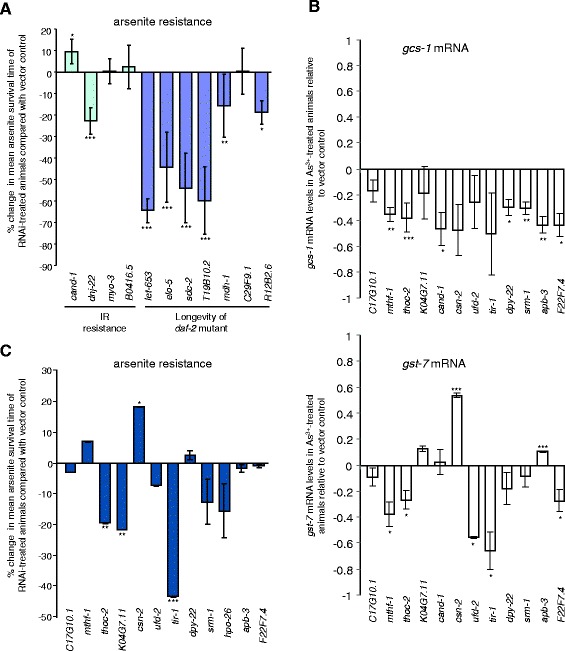
**Effect of selected candidate genes on arsenite-induced gene expression and arsenite resistance. (A)** The effect of selected RNAi targeting of genes required for resistance to ionizing radiation (IR) [18] or the extended lifespan of *daf-2* mutant [19] on arsenite resistance. **(B)** Twelve of the most robust regulators (Table 1) on arsenite-induced expression of *gcs-1* (upper panel) and *gst-7* (lower panel) and **(C)** arsenite resistance. In (A) and (C), comparisons are shown between the mean survival time following exposure of wild-type animals treated with the indicated RNAi or empty vector control to 7.5 mM arsenite. Mean survival times were calculated relative to control based on data obtained from a minimum of three separate experiments (see Additional file 2: Figure S4 for representative experiments). Error bars represent the standard error of the mean. Statistically significant differences between the survival of RNAi-treated and control animals (*P ≤ 0.05; **P ≤ 0.01; ***P ≤ 0.001) were identified by Cox’s regression analysis of the data obtained in all the experiments. In (B), comparisons are shown between the levels of *gcs-1* (upper panel) or *gst-7* (lower panel) mRNA in wild-type animals treated with the indicated RNAi or empty vector control following exposure to 5 mM arsenite for 30 min. Mean RNA levels relative to control were calculated from a minimum of three separate experiments. Error bars represent the standard error of the mean. Statistically significant differences between the *gcs-1* and/or *gst-7* mRNA levels in RNAi-treated and control animals (Student’s *T* test) are indicated (*P ≤ 0.05; **P ≤ 0.01; ***P ≤ 0.001). IR, ionizing radiation; RNAi, RNA interference.

Although the function of more than one-third of the identified genes is unknown, bioinformatic analysis revealed the remainder to represent a broad range of biological processes (see Additional file 2: Figure S2). To identify possible functional overlaps, we compared the results of our screen with published RNAi screens of the same RNAi library. This revealed a statistically significant overlap (*P* = 0.0039) between the genes we have identified and those found to be required for resistance to ionizing radiation (IR) (see Additional file 1: Table S4) [18]. This suggests that the role of these particular genes in IR resistance might be to up-regulate phase 2 detoxification gene expression to protect against free radicals that are generated by IR. A significant overlap (*P* = 0.0008) was also found between genes required for *gcs-1p::gfp* expression and those required for the increased lifespan of insulin receptor (*daf-2*) mutants (see Additional file 1: Table S4) [19]. This supports other studies that have suggested a positive correlation between the expression of phase 2 detoxification systems and lifespan [3,4]. Indeed, consistent with the vital role of *gcs-1* in arsenite resistance [20], six of the seven genes required for both the longevity of *daf-2* mutants [19] and intestinal *gcs-1p::gfp* expression were important for the resistance of wild-type animals to arsenite (Figure 2A). This suggests that, as well as the increased lifespan, these genes may be important for the increased stress resistance associated with reduced insulin signalling. However, of the four genes previously identified as required for resistance to IR [18], only *dnj-22* was also important for arsenite resistance (Figure 2A) and *cand-1* RNAi actually increased arsenite resistance (Figure 2A). This suggests that alternative mechanisms to promote stress resistance can compensate for the reduced induction of *gcs-1*.

### Identification of new genes required for arsenite-induced *gcs-1* expression and resistance to arsenite

Next we examined whether candidate regulators of *gcs-1* affected the expression of a second phase 2 transcriptional reporter gene, *gst-4p::gfp*, which is detectably expressed in the hypodermis/body wall muscle and intestine of wild-type animals under normal conditions [21]. Strikingly, of the 355 RNAi clones initially identified, only 24 reduced the intestinal level of *gst-4p::gfp* expression (see Additional file 1: Table S3). This provided further corroboration that genes identified by our screen are not generally required for intestinal gene expression or GFP stability. However, quantitative analysis of a subset of 24 candidates, identified 13 additional clones that significantly reduced intestinal *gst-4p::gfp* expression in *prdx-2* mutant animals (see Additional file 2: Figure S3). This suggests that approximately half of the genes identified as important for intestinal expression of *gcs-1*, might also be important for the elevated expression of other phase 2 genes in animals lacking PRDX-2. Notably, none of these RNAi clones prevented pharyngeal *gcs-1p::gfp* or hypodermal *gst-4p::gfp* expression. Although the effectiveness of RNAi may be reduced in the pharynx, this suggests that they are not required for housekeeping levels of phase 2 gene expression in other tissues.

Although identified RNAi clones were likely to include any that can rescue the defects of the *prdx-2* mutant, we predicted our screen to have also identified genes required for the increased intestinal expression of phase 2 detoxification genes under stress conditions, here mimicked by loss of the peroxidase PRDX-2 [14]. To test this hypothesis, we examined whether candidate genes were also required for the arsenite-induced expression of *gcs-1p::gfp* in wild-type animals. Although high throughput RNAi screens for *C. elegans* have intrinsically high false negative rates [22], we reasoned that less reproducible effects were also likely to indicate RNAi clones with more marginal effects on intestinal *gcs-1p::gfp.* Hence, we chose the 90 most robust RNAi clones that ablated intestinal *gcs-1p::gfp* expression in *prdx-2* mutants in each of four separate screenings (see Additional file 1: Table S3). Importantly, the reproducibility of these clones also indicates that the RNAi treatment was consistently effective. Excitingly, more than half of these RNAi clones (53) prevented arsenite-induced, intestinal *gcs-1p::gfp* expression in at least one of three trials, indicating that our screen had successfully identified new candidate activators of arsenite-induced phase 2 gene expression (see Additional file 1: Table S5). As the most robust of these candidates, the 16 RNAi clones that prevented arsenite-induced, intestinal *gcs-1p::gfp* expression in each of three trials, were selected for further analysis (Figure 1 and Table 1).

**Table 1:** **Activators of stress-induced** ***gcs-1p::gfp*** **expression and their effects on stress-induced phase 2 gene expression, stress resistance, PMK-1 and SKN-1**

**Functional classification of gene targeted by RNAi**			**Intestinal** ***gst-4p::gfp*** **expression**		**mRNA (Figure** 2 **B)**		**Arsenite resistance**	**PMK-1 activation**	**Nuclear levels of SKN-1** ^**S393A**^ **::GFP**
			**WT (N2) (Additional file** **1** **: Table S3)**	***prdx-2*** **(Additional file** **2** **: Figure S3)**	***gcs-1***	***gst-7***	**(Figure** 2 **C)**	**(Figure** 4 **)**	**(Figure** 6 **A)**
**Gene expression**	**Human orthologue**	**Proposed function**							
*C17G10.1*	*OGFOD1*	Regulates translation	-	-	↓	-	-	↑	-
*C35C5.1 sdc-2*	*GOLGA6L5*	Sex determination/dosage compensation	↓	ND	ND	ND	↓***	↑	*-*
*F47A4.2 dpy-22*	*MED12*	Mediator subunit	-	-	↓*	↓	-	↑	-
*C16A3.8 thoc-2*	*THOC2*	Transcriptional elongation	-	↓***	↓***	↓*	↓**	↑	↓***
*K04G7.11*	SYF2	Pre-mRNA-splicing factor	-	↓**	↓	↑	↓	↑	↑***
Protein homeostasis									
*Y102A5A.1 cand-1*	*CAND1*	Cullin-associated NEDD8-dissociated	-	↓***	↓*	*-*	↑*	↑	-
*B0025.2 csn-2*	*CSN2*	Cop9 signalosome subunit	↑	↓***	↓	↑***	↑*	-	↑***
*T05H10.5 ufd-2*	*UBE4B*	E4 ubiquitin conjugating enzyme	↓	↓***	↓	↓*	↓	-	↓***
Signal transduction									
*F13B10.1 tir-1*	*SARM1*	Pathogen responses	-	↓***	↓	↓*	↓***	↓***	-
*T28H11.2 srm-1*	-	Serpentine receptor	-	-	↓**	-	↓	↑	-
*ZK792.3 inx-9*	*-*	Innexin	*-*	*-*	ND	ND	ND	ND	*-*
Growth/metabolism									
*C06A8.1 mthf-1*	*MTHFR*	Methylenetetrahydrofolate reductase	-	↓***	↓**	↓*	↓	↑	-
*C34F11.3*	*AMPD2*	AMP deaminase 2	*-*	↓***	ND	ND	ND	ND	-
Structural									
*F46E10.11 hpo-26*	-	Hypersensitive to pore-forming toxin	-	↓***	ND	ND	↓	↑	↑***
Transport/trafficking									
*R11A5.1 apb-3*	AP3B1/2	Adaptin	-	-	↓**	↑***	-	↑	↑***
Unknown function									
*F22F7.4*	-		-	-	↓*	↓*	-	-	-

Sixteen RNAi that prevented any detectable intestinal *gcs-1p::gfp* expression in *prdx-2* mutant animals and in more than 50% of wild-type animals treated with 1 mM arsenite for 90 min (N2 *gcs-1p::gfp*) on each of three occasions on which they were screened, following quantitative analysis, but which did not reduce intestinal expression of a non-phase 2 reporter gene, *F09E5.3::gfp* (Additional file 1: Table S3). These genes were placed into categories based on gene ontology [15]. Where RNAi affected *gst-4p::gfp* expression in wild-type (Additional file 1: Table S3) or *prdx-2* mutant animals (Additional file 2: Figure S3) the effect is indicated by an arrow. Where RNAi produced a greater than 10% change in *gcs-1* or *gst-7* mRNA levels in arsenite-treated animals (Figure 2B), arsenite resistance (Figure 2A,C), PMK-1 phosphorylation in arsenite-treated animals (Figure 4) or levels of SKN-1^S393A^::GFP in intestinal nuclei (Figure 6A) this is indicated. Statistically significant differences from control animals are indicated (*P < 0.05; **P < 0.01; ***P < 0.001). ND, not determined; RNAi, RNA interference; WT, wild type.

First, we examined whether these 16 potential new activators of arsenite-induced *gcs-1* expression were required for arsenite-induced increases in endogenous *gcs-1* expression. Analysis of the *gcs-1p::gfp* reporter line suggests that the constitutive, pharyngeal expression, which is unaffected by these clones, is likely to make a substantial contribution to total *gcs-1* mRNA levels. Nevertheless, the increased intestinal expression of *gcs-1* following arsenite treatment is reflected in an approximately 2.5-fold increase in total *gcs-1* mRNA [8]. Hence, we examined whether RNAi clones targeting a subset of 12 genes, representative of each functional category (Table 1), affected the total levels of *gcs-1* mRNA in arsenite-treated animals. These RNAi clones all reduced total *gcs-1* mRNA levels in arsenite-treated wild-type animals, with seven out of twelve clones producing a statistically significant decrease. In many cases, *gcs-1* mRNA levels were reduced to 40% to 50% of vector control levels, approximating *gcs-1* mRNA levels in untreated animals and thus indicating that induction by arsenite was greatly impaired (Figure 2B upper panel). Thus mRNA analysis is consistent with the reporter screens, strongly suggesting that the majority of genes in Table 1 are important for the arsenite-induced expression of *gcs-1* (Figure 2B upper panel). Notably, only ten of these sixteen RNAi’s reduced the intestinal expression of *gst-4p::gfp* in either wild-type or *prdx-2* mutant animals (Table 1, Additional file 1: Table S3 and Additional file 2: Figure S3). Hence, to further test the generality of the role of candidate genes in phase 2 gene expression, we investigated whether the subset of 12 RNAi affected the arsenite-induced expression of another phase 2 gene, *gst-7*, which is also induced two- to threefold by arsenite [8]. This revealed that RNAi targeting five of these genes (*tir-1*, *ufd-2*, *thoc-2*, *mthf-1* and *F22F7.4*) significantly reduced *gst-7* mRNA levels in arsenite-treated animals (Figure 2B lower panel). However, RNAi targeting of other candidate genes either had little effect, or in two cases, *csn-2* and *apb-3*, actually significantly increased *gst-7* mRNA levels (Figure 2B lower panel). Together with our reporter analysis (Table 1, see Additional file 2: Figure S3), this suggests that while some genes are more broadly required for stress-induced phase 2 detoxification gene expression, others may be specifically required for the stress-induced expression of *gcs-1* (Figure 2B,C).

**Figure 3:**
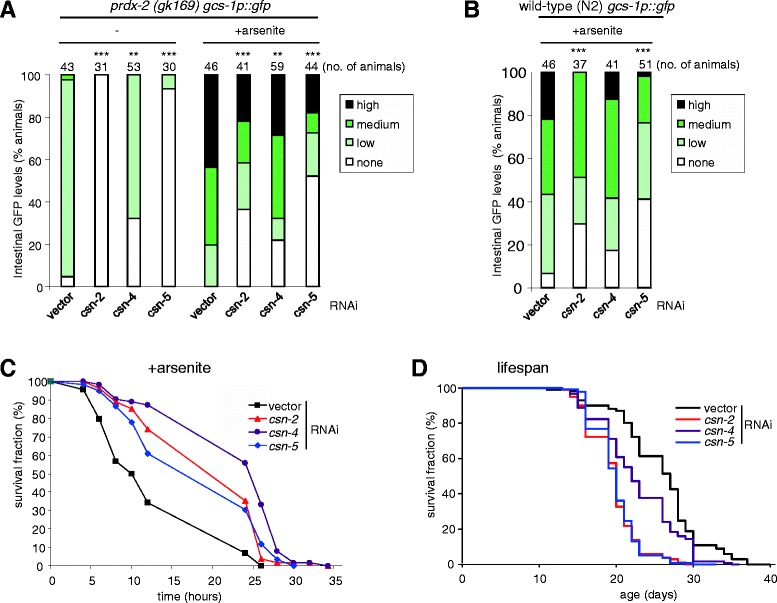
**Cop9 signalosome has multiple roles in the regulation of** ***gcs-1p::gfp*** **expression, arsenite resistance and longevity.** *csn-2*, *csn-4* and *csn-5* RNAi significantly **(A)** reduce the basal and arsenite-induced intestinal expression of *gcs-1p::gfp* in *prdx-2* mutant animals, **(B)** reduce the arsenite-induced expression of *gcs-1p::gfp* in wild-type animals, **(C)** increase the arsenite resistance but **(D)** reduce the lifespan of wild-type animals compared with animals maintained on empty vector control. In (A) and (B), levels of intestinal GFP were assessed under the fluorescent stereoscope using the scoring criteria illustrated in Figure 1 and described in Methods. Statistically significant differences between RNAi-treated samples and empty vector control are indicated. **P < 0.01; ***P < 0.001. (C) The survival on 7.5 mM arsenite and (D) lifespan of *csn-2*, *csn-4* and *csn-5* RNAi were statistically significant from empty-vector-control-treated animals (*P* < 0.001 in each case). Each experiment was repeated at least twice with similar results. GFP, green fluorescent protein; RNAi, RNA interference; WT, wild type.

Next we examined whether these 12 genes were important for arsenite resistance. Notably, RNAi targeting of four genes (*sdc-2*, *thoc-2*, *K04G7.11* and *tir-1*) significantly increased the sensitivity to arsenite toxicity (Figure 2A,C and see Additional file 2: Figure S4). However, in most cases arsenite resistance was unaffected or only slightly reduced (Figure 2C). Moreover, similar to *cand-1* RNAi (Figure 2A), RNAi targeting of a second regulator of protein homeostasis, *csn-2*, actually increased arsenite resistance (Figure 2C). This contrasted with the effects of other RNAi clones identified by our screen, but was consistent with the increased intestinal *gst-4p::gfp* and *gst-7* mRNA levels observed in *csn-2* RNAi-treated animals (Table 1 and see Additional file 1: Table S3) and the previously reported role of CSN-2 in regulation of phase 2 detoxification genes and peroxide resistance [17]. CSN-2 is a component of the Cop9 signalosome (CSN), which, as a multisubunit complex, influences protein turnover by removing the ubiquitin-like modifier NEDD8 from Cullin-Ring ubiquitin ligases [23]. In agreement with these previous studies, our data suggest that CSN-2, and other CSN subunits, suppress basal phase 2 gene expression under normal growth conditions (Table 1) but are required for stress-induced increases in *gcs-1* expression (e.g., arsenite or loss of *prdx-2*) (Table 1, Figure 3A,B). Notably, although *gcs-1* induction is impaired in CSN-deficient animals (Figures 3A,B, 2B upper panel), the high basal *gcs-1* expression and increased expression of other phase 2 detoxification genes, e.g., *gst-7* (Figure 2B lower panel, see Additional file 2: Figure S5), is sufficient to confer increased oxidative stress resistance (Figures 2C and 3C) [17]. This suggests that for survival following exposure to an acutely toxic level of arsenite, basal levels of phase 2 detoxification gene expression may be more important than the ability to induce *gcs-1* (Figure 2). Strikingly, despite their increased resistance to acute oxidative stress, under normal growth conditions CSN-deficient animals were short-lived (Figure 3D).

### The role of genes required for arsenite-induced *gcs-1p::gfp* expression in arsenite-induced activation of PMK-1

**Figure 4:**
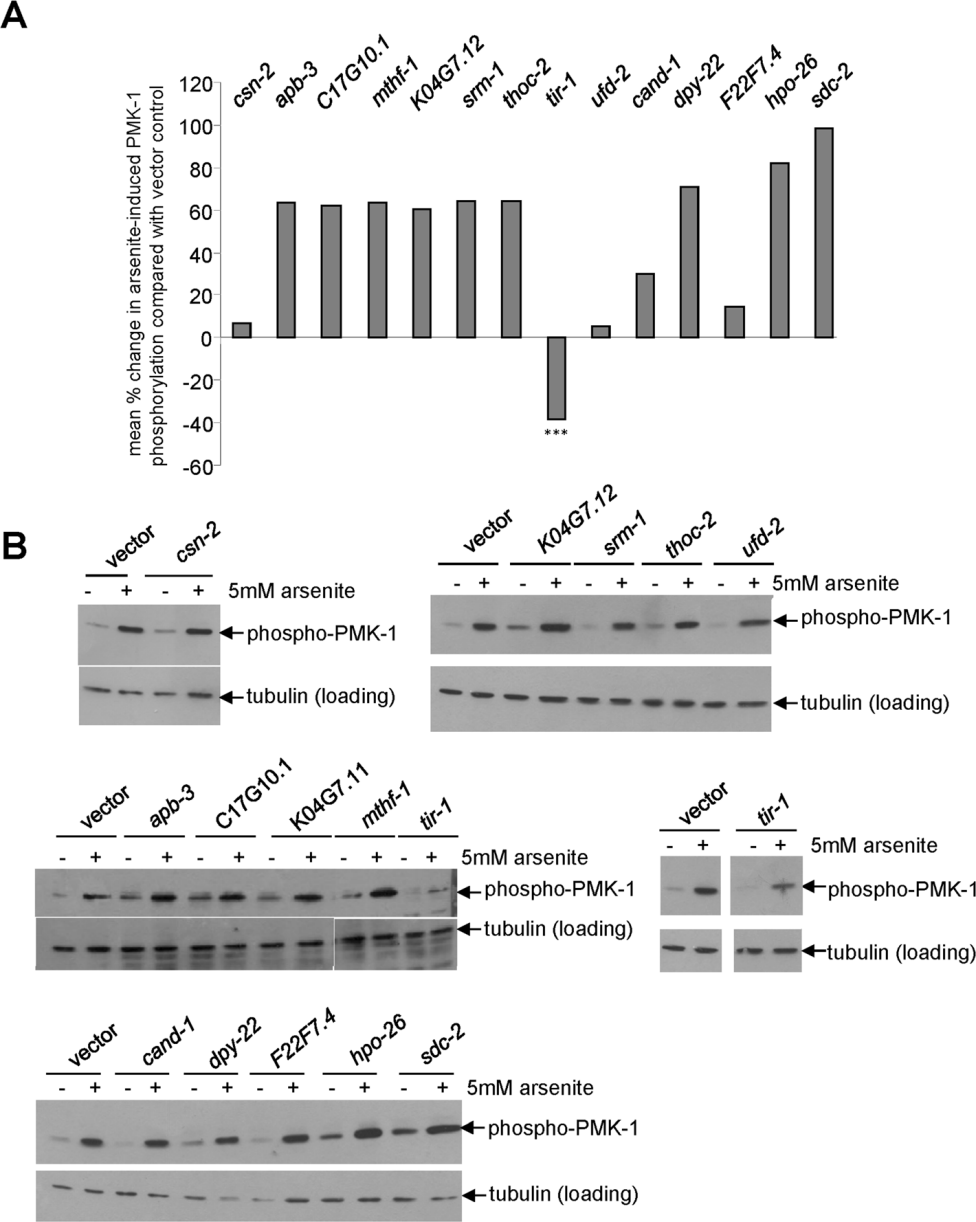
**Effect of genes required for stress-induced activation of** ***gcs-1*** **on arsenite-induced PMK-1 activation.** Western blot analysis of RNAi and vector-control-treated animals before and following 5 min exposure to 5 mM arsenite revealed that out of 14 of the most robust activators of *gcs-1p::gfp* (Table 1), only *tir-1* RNAi reduced the level of PMK-1 phosphorylation. **(A)** Mean percentage difference between the level of PMK-1 phosphorylation in arsenite-treated control and RNAi-treated animals following analysis of quantitative densitometry data obtained from at least two independent experiments. ***indicates that *tir-1* RNAi significantly reduced PMK-1 phosphorylation compared with vector control (Student’s *T* test, *P* = 0.00056). The effects of other RNAi clones on arsenite-induced PMK-1 phosphorylation were not statistically significant (*P* > 0.05). **(B)** Representative Western blots of those quantitatively analysed in (A). RNAi, RNA interference.

Having established that candidate genes were required for arsenite-induced *gcs-1* expression (Figure 2B upper panel and see Additional file 1: Table S3) but had different effects on the arsenite-induced expression of other phase 2 genes (Figure 2B lower panel and see Additional file 2: Figure S3) and/or arsenite resistance (Figure 2A,C), we proceeded to investigate the mechanisms by which selected candidate genes (Table 1) promoted stress-induced *gcs-1* expression. First, we examined whether these genes contributed to known mechanisms involved in arsenite-induced phase 2 gene expression. An important response to arsenite is the activation of PMK-1 by SEK-1-mediated phosphorylation. Following activation, PMK-1 phosphorylates SKN-1 increasing nuclear SKN-1 levels and promoting *gcs-1* expression [11]. Hence, we tested whether genes required for arsenite-induced activation of *gcs-1p::gfp* (Table 1) were required for PMK-1 activation. Strikingly, only RNAi targeting the Toll/interleukin-1 receptor domain protein, TIR-1, reduced the arsenite-induced activation of PMK-1 (Figure 4). This suggests that the other genes are required for alternative, PMK-1-independent, mechanisms to activate *gcs-1p::gfp*. Indeed, there was even an indication that many RNAi clones caused an increase in PMK-1 phosphorylation, perhaps reflecting a compensatory activation of PMK-1 when other signalling pathways are disrupted (Figure 4).

### TIR-1 and NSY-1 are required for arsenite-induced activation of the p38-related MAPK, PMK-1

**Figure 5:**
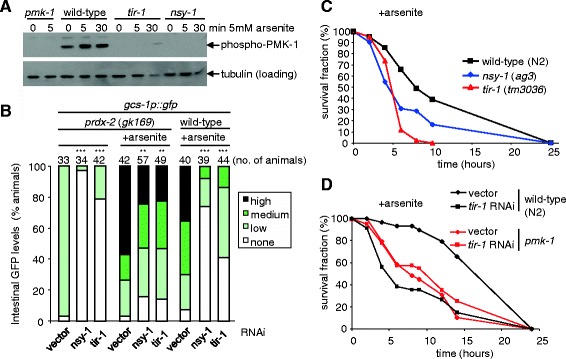
**TIR-1 and NSY-1 are required for responses to arsenite. (A)** Western blot analysis of the phosphorylation of PMK-1 in wild-type (N2), *tir-1* (*tm3036*), *nsy-1* (*ag3*) and *pmk-1* (*km25*) mutant animals before and following treatment for the indicated times with 5 mM sodium arsenite. This revealed that TIR-1 and NSY-1 are both required for the phosphorylation of PMK-1, which occurs maximally within 5 min of arsenite treatment. β-tubulin levels are shown as a loading control. **(B)** The basal and arsenite-induced intestinal expression of *gcs-1p::gfp* is reduced following *tir-1* and *nsy-1* RNAi treatment of *prdx-2* mutant and in wild-type animals treated with arsenite. *** indicates a statistically significant difference from vector control *P* < 0.001; ** indicates a statistically significant difference from vector control *P* < 0.01 (chi^2^ test). **(C)** *tir-1* (*tm3036*) and *nsy-1* (*ag3*) mutant animals are significantly more sensitive to 10 mM arsenite than wild-type (N2) (log-rank N2 vs *tir-1*, *P* < 0.001; N2 vs *nsy-1*, *P* = 0.004). All experiments were repeated at least twice with similar results. **(D)** Assessment of the viability of *tir-1* RNAi and vector-control-treated wild-type and *pmk-1* mutant animals on 7.5 mM arsenite revealed that *tir-1* RNAi increases the arsenite-sensitivity of wild-type (N2) but not *pmk-1* (*km25*) animals. Statistical analysis: wild-type vector vs *tir-1* RNAi, *P* <0.001; *pmk-1* vector vs *tir-1* RNAi, *P* = 0.32; wild-type vector vs *pmk-1* vector, *P* < 0.001. GFP, green fluorescent protein; RNAi, RNA interference.

Previous studies have shown that TIR-1 and the MAPKKK NSY-1 are both required for the activation of PMK-1 in response to pathogenic infection but not for responses to arsenite [11,24,25]. Hence, to confirm our RNAi data suggesting that TIR-1 is required for arsenite-induced activation of PMK-1 (Figure 4), we examined the phosphorylation of PMK-1 in wild-type, *tir-1* and *nsy-1* mutant *C. elegans*. Consistent with previous studies, treatment of wild-type animals with 5 mM arsenite caused a rapid increase in the level of PMK-1 phosphorylation that was maximal by 5 min (Figure 5A) [14]. In contrast, there was no detectable phosphorylation of PMK-1 in *tir-1* or *nsy-1* mutant *C. elegans* after 5 min arsenite treatment. This suggests that TIR-1 and NSY-1 are both required for the arsenite-induced activation of PMK-1. Indeed, although *nsy-1* was not identified by our initial genome-wide screen, both *tir-1* and *nsy-1* RNAi significantly reduced the level of *gcs-1p::gfp* induced by arsenite in wild-type and *prdx-2* mutant animals (Figure 5B). Moreover, consistent with the important role of PMK-1 activation and *gcs-1* expression in resistance to arsenite [11,20], *tir-1* and *nsy-1* mutant animals were significantly more sensitive to arsenite toxicity than wild-type animals (Figure 5C). Thus, here we have identified that TIR-1 and NSY-1, which are required for PMK-1 activation during infection, are also important for activation of PMK-1 in response to arsenite. Importantly, the inability of *tir-1* RNAi treatment to increase further the arsenite-sensitivity of *pmk-1* mutant animals (Figure 5D) suggests impaired PMK-1 activation underlies the arsenite-sensitivity of TIR-1-deficient animals (Figure 5).

### Candidate RNAi that reduces levels of SKN-1 in intestinal nuclei has broad effects on arsenite-induced phase 2 detoxification gene expression

**Figure 6:**
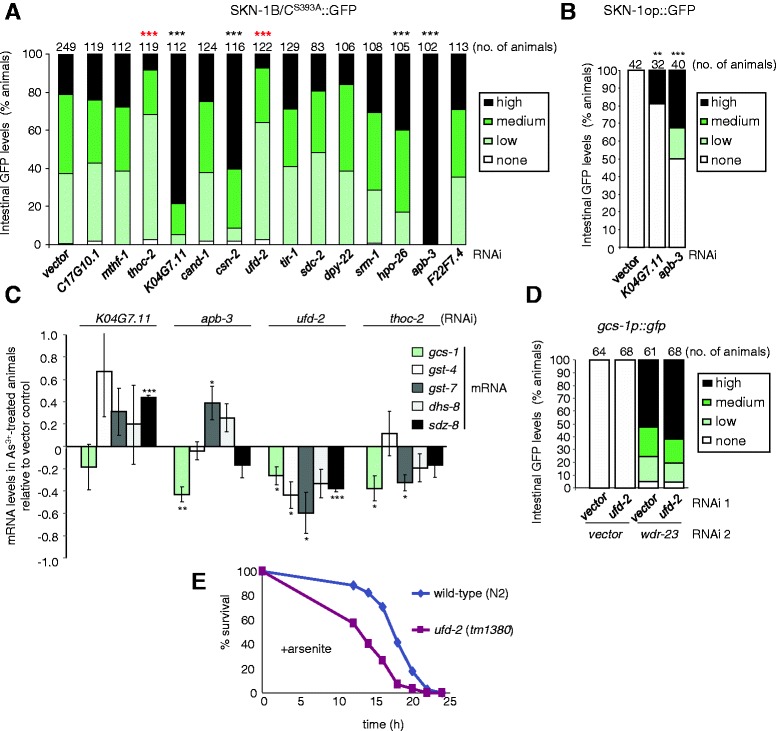
**Activators of** ***gcs-1*** **have diverse effects on SKN-1 and expression of other SKN-1 target genes.** Some candidate activators act independently or downstream from SKN-1 to increase *gcs-1* expression whereas others, such as UFD-2, are required for nuclear SKN-1 levels and arsenite-induced expression of multiple phase 2 detoxification genes. **(A)** The effect of 14 RNAi that reduce intestinal *gcs-1p::gfp* expression in *prdx-2* mutant and arsenite-treated animals (Table 1) on the levels of a constitutively active form of SKN-1 (SKN-1B/C^S393A^::GFP) present in intestinal nuclei [12]. RNAi that produced a statistically significant decrease (***P < 0.001) or increase (***P < 0.001; *P < 0.05) in nuclear SKN-1 levels are indicated. The data shown were obtained in three independent experiments. **(B)** *K04G7.11* and *apb-3* RNAi significantly increased (***P < 0.001; **P < 0.01) wild-type SKN-1::GFP forms (SKN-1op::GFP) [4] in two independent experiments (representative experiment is shown). **(C)** Effect of *K04G7.11*, *apb-3*, *ufd-2* and *thoc-2* RNAi, compared with vector control, on the total *gcs-1*, *gst-4*, *gst-7*, *dhs-8* and *sdz-8* mRNA levels in animals treated for 30 min with 5 mM sodium arsenite. **(D)** *wdr-23* RNAi causes a similar increase in intestinal expression of *gcs-1p::gfp* in vector control and *ufd-2* RNAi-treated animals. The data shown were obtained in two independent experiments. **(E)** The survival of L4-larval-stage wild-type and *ufd-2* (*tm1380*) mutant animals on plates containing 7.5 mM sodium arsenite (As^3+^) was monitored with log-rank analysis indicating that *ufd-2* (*tm1380*) mutant animals are significantly more sensitive to the toxicity of arsenite than wild type (N2) (*P* < 0.001 in each case). *n* = 30 to 36.

Next, we focused on determining how other candidate genes promoted arsenite-induced *gcs-1* expression. The arsenite-induced expression of *gcs-1* is highly dependent on the SKN-1 transcription factor [8]. Although PMK-1-dependent phosphorylation is important for accumulation of SKN-1 in intestinal nuclei, SKN-1 activity is also regulated by other mechanisms. For instance, phosphorylation of SKN-1 by GSK-3 or the insulin-regulated AKT/SGK kinases directly inhibits SKN-1 activity [4,12]. Accordingly, we investigated whether other genes identified by our screen might be required for the activation of SKN-1 by a PMK-1-independent mechanism. As the basal levels of SKN-1::GFP in intestinal nuclei are normally undetectably low, to test this hypothesis we examined whether candidate genes (Table 1) affected the nuclear abundance of a constitutively active form of SKN-1 in which the GSK-3 phosphorylation site had been mutated; SKN-1^S393A^::GFP [12]. Unexpectedly, RNAi targeting several genes, including *K04G7.11* and *apb-3*, substantially *increased* nuclear SKN-1^S393A^::GFP levels (Figure 6A). Moreover, whereas wild-type SKN-1::GFP was undetectable in the intestinal nuclei of control animals, these RNAi clones also caused wild-type SKN-1::GFP to be detected in the intestinal nuclei of a significant number of animals (Figure 6B). Thus, paradoxically, loss of *K04G7.11* and *apb-3* prevents arsenite-induced increases in intestinal *gcs-1p::gfp* expression (Table 1) and *gcs-1* mRNA levels (Figure 2B), despite increased levels of nuclear SKN-1 (Figure 5A). This suggests both genes act downstream of SKN-1, or through parallel SKN-1-independent pathways, to promote *gcs-1* expression. Notably, *apb-3* and *K04G7.11* RNAi did not prevent the arsenite-induced expression of four other phase 2 genes, *gst-7*, *gst-4*, *dhs-8* and *sdz-8* (Figure 2B and 6C). Indeed, consistent with the increased levels of nuclear SKN-1, *gst-7*, *gst-4*, *dhs-8* and *sdz-8* mRNA levels were even slightly increased in *apb-3* and *K04G7.11* RNAi-treated animals (Figure 6C). This indicates *apb-3* and *K04G7.11*, like *csn-*2, and potentially other genes identified by our screen (Figure 6A), are not universally important for the arsenite-induced expression of all phase 2 genes but specifically required for arsenite-induced increases in *gcs-1* mRNA expression.

Although the reductions in PMK-1 activity associated with *tir-1* RNAi (Figure 4) were insufficient to inhibit nuclear accumulation of the hyperactive SKN-1^S393A^GFP mutant (Figure 6A), excitingly, RNAi clones targeting *thoc-2* and *ufd*-*2* significantly reduced the levels of SKN-1^S393A^::GFP in intestinal nuclei, indicating that UFD-2 and THOC-2 are required for nuclear SKN-1 abundance (Figure 6A). SKN-1 activity is essential for the *C. elegans* transcriptional response to arsenite [8]. Indeed, in contrast to *apb-3* and *K04G7.11*, *thoc-2* and *ufd-2* were important, not only for the arsenite-induced expression of *gcs-1*, but of several other SKN-1-regulated phase 2 genes (Figure 6C). Indeed, *ufd-2* RNAi produced a statistically significant reduction in the total mRNA levels for all five phase 2 genes that we investigated.

To test whether the associated reduction in SKN-1 activity could underlie the reduced arsenite-induced phase 2 gene expression in these UFD-2-deficient animals (Figure 6C), we examined whether *wdr-23* RNAi, which increases SKN-1 activity and intestinal expression of *gcs-1p::gfp* (Figure 6D) [13,26], bypassed the requirement for UFD-2. The inability of *ufd-2* RNAi to reduce intestinal *gcs-1p::gfp* expression in *wdr-23* RNAi-treated animals (Figure 6D) suggests that loss of WDR-23 increases SKN-1 levels sufficiently to restore normal expression of phase 2 genes to UFD-2-deficient animals. These data are consistent with UFD-2 promoting arsenite-induced phase 2 gene expression and oxidative stress resistance by increasing SKN-1 activity. Moreover, despite normal levels of PMK-1 phosphorylation, but consistent with the reduced SKN-1 activity and arsenite tolerance associated with *ufd-2* RNAi, *ufd-2* (*tm1380*) mutant animals (predicted null) were also more sensitive to arsenite toxicity than wild-type animals (Figure 6E and see Additional file 2: Figure S6).

## Discussion

**Figure 7:**
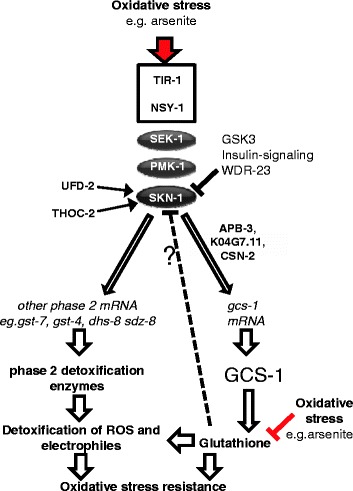
**Proposed model indicating the different roles of candidate activators in the activation of phase 2 detoxification gene expression** **.** TIR-1, NSY-1, THOC-2, UFD-2, K04G7.11, APB-3 and CSN-2 participate in the activation of phase 2 detoxification gene expression and oxidative stress resistance. In *C. elegans* the SKN-1 transcription factor is essential for phase 2 detoxification gene expression and oxidative stress resistance. Levels of SKN-1 in intestinal nuclei are normally low due to inhibitory phosphorylation by (a) GSK-3 (serine 393) [12] and (b) insulin-regulated kinases [4] and (c) interaction with WDR-23, which inhibits DNA binding [13,26]. Oxidative stress increases the phosphorylation of the PMK-1 MAPK. Active PMK-1 phosphorylates SKN-1, increasing levels of SKN-1 in intestinal nuclei, phase 2 gene expression and oxidative stress resistance [11]. Our data suggest that TIR-1 and NSY-1 are important for arsenite-induced activation of PMK-1 but that THOC-2 and UFD-2 act downstream of PMK-1 to promote SKN-1 activity by unidentified mechanism(s). Intriguingly, our data suggest that several candidates identified by our screen, including APB-3, CSN-2 and K04G7.11, may be dispensable for the expression of other phase 2 detoxification genes but act to increase expression of *gcs-1* via unidentified mechanisms downstream or independently from PMK-1 and SKN-1. We suggest that the increased nuclear levels of SKN-1 in *apb-3*, *csn-2* and *K04G7.11* RNAi-treated animals, which have lower arsenite-induced *gcs-1* expression (Figure 2B and 6C), could indicate that glutathione contributes to a feedback mechanism to inhibit SKN-1 (as indicated by ?). ROS, reactive oxygen species.

Conserved MAPK (p38 and PMK-1) and CnC transcription factors (Nrf2 and SKN-1) are vital for stress-induced increases in phase 2 detoxification gene expression and accordingly protect both normal and tumour cells from xenobiotic/drug-induced oxidative damage. Although work has focused on the regulation of p38 (PMK-1)/Nrf2 (SKN-1) activity, here we provide evidence that the regulation of stress-induced increases in phase 2 gene expression in animals is much more complex than previously appreciated. Our RNAi screening in *C. elegans* has uncovered new genes that are required for the elevated expression of a phase 2 detoxification gene (*gcs-1*) under stress conditions (Table 1 and see Additional file 1: Table S3). Partial characterization of some of the most robust candidate genes has identified new genes required for the arsenite-induced activation of PMK-1 and SKN-1. However, despite PMK-1's critical role in promoting arsenite-induced gene expression [8,11], our data suggest that many of these new activators of *gcs-1* expression participate in novel mechanisms to promote phase 2 gene expression that are downstream or independent of PMK-1 (Figure 7). For example, UFD-2 is required for SKN-1 activity but not PMK-1 activation (Figures 4B, and 6, see Additional file 2: Figure S6). Moreover, our screening has revealed genes, such as *mthf-1* and *F22F7.4*, that are important for arsenite-induced increases in the expression of multiple phase 2 genes without apparently affecting either PMK-1 or SKN-1 activity. This raises the intriguing possibility that a significant number of genes identified here may contribute to the stress-induced activation of phase 2 genes by mechanisms outside of the canonical PMK-1/SKN-1 pathway.

### Identification of new genes required for the activation of specific or multiple phase 2 genes

SKN-1 is generally important for the stress-induced expression of multiple phase 2 genes. Consistent with this, the new activators of PMK-1 and SKN-1 that we have identified, are important for the activation of multiple phase 2 genes (Table 1 and Figure 6). Indeed, like SKN-1 [8], UFD-2 is also required for intestinal *gst-4* expression under non-stress conditions (Table 1 and Additional file 2: Table S3). However, our data suggest that many of the genes identified here may be specifically important for the stress-induced expression of *gcs-1* (Figures 2B and 6C, and Table 1 and see Additional file 2: Figure S3)*.* Studies of yeast have revealed that transcriptional responses to oxidative stress involve the activation of parallel signalling pathways and transcription factors to coordinate the expression of distinct and overlapping sets of phase 2 genes [27]. Excitingly, our data suggest that alternative pathways, involving some of the genes identified here, may also contribute to the regulation of different subsets of phase 2 genes in animals. For instance, unexpectedly, we have identified several genes (e.g., *apb-3* and *csn-2*) that are required for stress-induced increases in *gcs-1* even in the presence of high levels of nuclear SKN-1. This suggests that arsenite-induced *gcs-1* expression also involves other regulators acting downstream of PMK-1 to promote SKN-1 activity specifically at this promoter. In addition to participating in the glutathionylation of toxic electrophiles, glutathione has a key role as a cell redox buffer. Hence, the presence of these additional regulatory mechanisms may be important for allowing *gcs-1* expression and glutathione biosynthesis to be regulated independently from other phase 2 genes (Figure 7).

### High basal levels of phase 2 detoxification gene expression protect against acute stress conditions but do not necessarily extend lifespan

RNAi targeting subunits of the CSN increase the expression of phase 2 reporter genes in wild-type animals [17]. Hence, we were surprised when our screen identified *csn-2* amongst the genes required for the elevated *gcs-1p::gfp* and *gst-4p::gfp* expression in the *prdx-2* mutant background. However, our further analysis has revealed that, despite high basal levels of phase 2 gene expression and increased levels of nuclear SKN-1 (Figure 6A) [17], CSN-deficient animals are unable to effectively increase the expression of *gcs-1* in response to arsenite (Figures 2B upper panel and 3A,B, and Table 1). Despite this attenuated response to arsenite, CSN-deficient animals are more resistant to both arsenite (Figure 2C and 3C) and another oxidative stress-causing agent, tert-butyl peroxide [17]. Indeed, analysis of other genes identified here indicates that the inability to induce *gcs-1* expression (Figure 2B upper panel), does not necessarily correlate with a lower arsenite tolerance (Figure 2C). Accordingly, we propose that in conditions of acute arsenite toxicity, basal levels of *gcs-1* expression may be more important for survival than the ability to increase *gcs-1* mRNA levels. This suggests that approaches to increase the basal levels of phase 2 gene expression may be effective as a means to prevent the acute toxicity associated with particular drugs [1].

Despite their increased oxidative stress resistance, CSN-deficient animals are short-lived (Figure 3D). This is consistent with increasing evidence that there is not necessarily a direct correlation between resistance to environmental oxidative stress and longevity. For example, *prdx-2* mutant animals have high basal expression of phase 2 detoxification genes and are more resistant to arsenite but age prematurely and are short-lived [14]. It is possible that the short lifespan of CSN-deficient and PRDX-2-deficient animals reflect unrelated functions of the CSN and PRDX-2. However, these findings do support other studies suggesting that constitutively high levels of phase 2 detoxification can be deleterious [4,14,28]. Thus, although increased SKN-1 activity can extend lifespan [4,13], the ability to regulate phase 2 detoxification gene expression in response to changes in the environment may also be important.

### TIR-1 and NSY-1 are required for PMK-1-dependent responses to oxidative stress and pathogens

Our analysis of the most robust regulators of *gcs-1* expression identified TIR-1 as important for arsenite-induced activation of the p38-related MAPK, PMK-1. TIR-1 and the MAPKKK NSY-1 are required for activation of PMK-1 in response to pathogens and for the resistance of *C. elegans* to infection [25]. Here we have shown that TIR-1 is also required for the induction of phase 2 detoxification gene expression and resistance to the oxidative stress-causing agent, arsenite. This indicates that arsenite and pathogens may activate similar signalling pathways/defence mechanisms and raises the intriguing possibility that the expression of phase 2 detoxification genes may contribute to the role of TIR-1 in protecting against pathogenic infection. This is consistent with recent studies revealing that SKN-1-dependent expression of phase 2 detoxification genes is important for *C. elegans* resistance to bacterial infection [29,30].

### Identification of genes that interact with *prdx-2*

Our screen has also uncovered genetic interactions with *prdx-2*. For instance, in the course of screening we identified 70 genes that may specifically be required for the development or reproduction of animals lacking PRDX-2 (see Additional file 1: Table S1). Consistent with a functional overlap between these genes and the peroxidase *prdx-2*, functional analysis revealed that many encoded stress defence proteins (Additional file 2: Figure S1). Indeed, the 11 RNAi clones that produced the most severe phenotype, preventing the survival or growth of *prdx-2* mutant *C. elegans*, included three targeting genes encoding peroxide-detoxifying enzymes: two catalases (*ctl-1* and *ctl-2*) and the mitochondrial 2-Cys peroxiredoxin (*prdx-3*). This suggests an essential role for peroxide-detoxifying enzymes that is partially redundant with *prdx-2* (see Additional file 1: Table S1).

Many of the RNAi clones that caused a reproducible loss of intestinal *gcs-1p::gfp* expression in *prdx-2* mutant worms did not affect the induction of *gcs-1p::gfp* by arsenite in wild-type animals. These will include any RNAi clones that specifically rescue the defects responsible for the elevated intestinal *gcs-1p::gfp* expression in *prdx-2* mutant animals or that are more specifically required for the induction of *gcs-1* in response to other stresses, e.g., peroxide. Further studies to distinguish between these possibilities may uncover stress-specific pathways to up-regulate phase 2 detoxification genes. Alternatively, by identifying genes required for Prx-specific effects on signalling, this may improve our understanding of how signalling functions contribute to the conserved role of Prx in longevity [31].

### Genes identified by this screen may include potential therapeutic targets for prevention or treatment of cancer and age-associated diseases

The important role that phase 2 detoxification enzymes play in protecting against drug and stress-induced cell damage has stimulated interest in developing ‘chemopreventive’ agents to increase their levels and prevent the toxic and carcinogenic effects associated with this oxidative damage. Conversely, phase 2 detoxification enzymes afford tumour cells important protection against chemotherapeutic agents, including arsenic (metalloid)-based drugs [5]. Our study provides important insight into the genes and pathways involved in up-regulating one of these enzymes in *C. elegans*. The conservation of stress response pathways suggests that human orthologues of these genes may provide new targets for approaches to manipulate the levels of phase 2 enzymes, to either protect cells or potentiate the toxicity of chemotherapeutic drugs. For instance, the human orthologue of UFD-2, UBE4B, has been proposed as a new target for chemotherapy, following the discovery that UBE4B promotes p53 degradation [32]. If the function of UFD-2 is conserved and UBE4B is important for Nrf2 activity, this raises the possibility that increased levels of phase 2 detoxification enzymes may also contribute to the survival of cancer cells expressing high levels of UBE4B. This would also indicate potential unwanted side effects of inhibition of UBE4B, including increased risk of adverse drug reactions.

In addition to the implications for chemotherapeutic interventions, our study supports the notion that the ability to up-regulate these defences in response to stress is important for longevity. As such it also provides new avenues to explore in the quest to delay or prevent the onset of age-associated diseases.

## Conclusions

The regulation of the expression of phase 2 detoxification enzymes in animals is more complex than previously appreciated, involving both general and gene-specific mechanisms.

## Methods

### *Caenorhabditis elegans* strains

All strains were maintained at 15°C using standard methods [33]. N2 Bristol (wild-type), VE1: *prdx-2*(*gk169*) II, LD1172: N2 *ldIs003* [*gcs-1p::gfp*], VE4: *prdx-2*(*gk169*) *ldIs003* [*gcs-1p::gfp*] II, BC14910 *dpy-5*(*e907*)/*dpy-5*(*e907*); *sEx14910* [*rCesF09E5.3::GFP + pCeh361*], CL2166: N2 *dvIs19* [pAF15(*gst-4p::gfp-nls*)] III, VE12: *prdx-2*(*gk169*) *dvIs19* [pAF15(*gst-4p::gfp-nls*)] III, AU3: *nsy-1*(*ag3*) II, IG685: *tir-1*(*tm3036*) III, KU25: *pmk-1*(*km25*) IV, PP198 *ufd-2*(*tm1380*) II, LD1257: N2 *ldEx010* [*SKN-1op::GFP*], LD1255: N2 *ldEx014* [*SKN-1op*^*S12A*^*::GFP*] and LD1252: N2 *ldEx020* [*SKN-1B/C*^*S393A*^*::GFP*].

### RNA interference

RNAi experiments were carried out essentially as described previously [34]. RNAi clones were grown in Luria Broth (LB) liquid media containing 50 μg/ml ampicillin overnight then diluted to OD_600_ = 1.0 and induced with 1 mM isopropyl β-D-1-thiogalactopyranoside (IPTG). This was used to seed plates, which were left at room temperature for 2 days to induce double-stranded RNA synthesis before worms were added.

### Genome-wide RNAi screening

Genome-wide RNAi screening was performed using a commercially available RNAi feeding library (MRC Geneservice, Cambridge, UK) [34]. Single colonies were inoculated into 800 μl of LB media containing 50 μg/ml ampicillin in 96-well deep well plates, covered with sterile, breathable film and incubated at 37°C on a shaking platform overnight. Then 1 mM IPTG was added to each well and bacteria harvested by centrifugation at 4°C, resuspended in 500 μl LB containing 100 μg/ml ampicillin and 1 mM IPTG then 20 μl spotted onto individual wells of 24-well plates containing RNAi agar. RNAi plates were incubated for 2 days at room temperature, then approximately ten synchronized L1-stage animals dispensed into each well. After incubation at 15°C for 7 days, larval stage F1 progeny were scored for intestinal GFP fluorescence using a Discovery V8 Zeiss fluorescent stereomicroscope. For each batch of RNAi clones tested, empty vector (pL4440) was included as a negative control. Clones that gave rise to less than approximately 40 viable progeny were scored lethal or sterile, as appropriate, and excluded from analysis of reporter gene expression. Clones that affected reporter expression were identified by comparing intestinal GFP fluorescence with empty vector controls. All RNAi clones that reduced intestinal *gcs-1p::gfp* expression to undetectable levels, i.e. ‘none’ (Figure 1), in more than 50% of the *prdx-2* mutant worms in the initial screen were re-screened three more times and also screened three times in wild-type animals for loss of *gst-4p::gfp*. For analysis of arsenite-induced intestinal *gcs-1p::gfp* expression, after 7 days of incubation on RNAi plates, worms were washed off screen plates in M9 buffer into the corresponding well of a 24-well plate in which the RNAi agar contained 1 mM sodium arsenite (Sigma S-7400, Poole, UK). Worms were incubated at 15°C for 90 min then reduced intestinal *gcs-1p::gfp* expression compared with empty vector control was assessed as described above. All RNAi clones that were analysed further were sequenced to confirm the identity of the targeted gene.

### Comparative analysis of genes identified in different genome-wide RNA interference screens

The statistical significance of the observed overlap between different gene lists obtained in different genome-wide RNAi screens was calculated using cumulative hypergeometric probability [35].

### Scoring of green fluorescent protein reporter gene expression

Intestinal expression of GFP reporter genes was scored similarly to that previously described [12]. ‘None’ indicates that no GFP was detected in intestinal cells, ‘low’ indicates that GFP was detected in the nuclei of a few (≤7) anterior or posterior intestinal cells, ‘medium’ indicates that GFP was detected in some (>7) but not all intestinal cells throughout the length of the intestine and ‘high’ indicates that GFP was detected in all cells throughout the intestine (see Figure 1 for representative images and illustrations). For more sensitive analysis of the effect of specific RNAi clones on intestinal GFP expression (in Table 1, Additional file 2: Figures S3 and S5 and Figure 5), animals were mounted on an agarose pad and scored under the 40× objective on a Zeiss Axioskop. Statistically significant differences between groups (P values) were determined using a chi^2^ test (Microsoft Excel).

### Arsenite resistance assays

Five to ten L4-stage animals were transferred to plates seeded with the indicated RNAi bacterial clone. Then 30 to 40 L4-stage F1 progeny of RNAi-treated animals (or OP50-maintained N2 and *ufd-2* (*tm1380*) mutants) were transferred to plates containing the indicated concentration of sodium arsenite and incubated at 15°C. Viability was assessed at the indicated time points and animals were scored as dead and removed from the plate if pharyngeal pumping had ceased and they did not respond to gentle prodding with a platinum wire. *P* values were derived from log-rank survival analyses of individual experiments (Minitable 16) or Cox’s regression analysis of multiple experiments (Figure 2).

### Analysis of lifespan

To analyse lifespan, 30 L4-stage hermaphrodites were placed onto the appropriate RNAi plate and allowed to lay eggs. Once the eggs developed to L4 stage, approximately 150 F1 progeny were transferred to fresh RNAi plates, so that there were 50 animals per plate. Once at the young adult stage, animals were transferred to RNAi plates containing 25 μM 5-fluoro-2'-deoxyuridine to prevent egg laying. Animals were transferred to new RNAi plates every few days throughout the experiment. Animals were incubated at 15°C and viability was assessed at least every 2 days at the same time of day. Animals were scored as dead and removed from the plate if pharyngeal pumping had ceased and they did not respond to gentle prodding with a platinum wire. Animals that died from bagging, ruptured vulva or crawled off the plate were censored. For statistical comparisons between control and RNAi-treated animals, *P* values were derived from a log-rank survival test (Minitab 16).

### Immunoblotting

Approximately 3,000 synchronized L1-larval-stage wild-type/mutant or control/RNAi-treated worms were added to the appropriate plates and harvested at L4 larval stage, before or following treatment with 5 mM arsenite. As described previously [14], extracts were prepared and equal amounts of protein (coomassie) analysed using antibodies against the dual phosphorylated form of p38 (#9211, Cell Signaling Technology) to detect phosphorylated PMK-1 and anti-β-tubulin antibodies (E7, Developmental Studies Hybridoma Bank, Iowa City, Iowa) as a loading control. Quantitative densitometric analysis (area under the peak) of Western blot autoradiographs was conducted using ImageJ 1.44 to determine phosphorylated PMK-1 levels relative to tubulin loading for each lane. The level of phosphorylated PMK-1 in RNAi-treated or mutant samples was then determined relative to control samples on the same blot.

### Analysis of *gcs-1* and *gst-7* mRNA expression

Wild-type (N2) animals were transferred to RNAi or control plates and RNA was extracted from approximately 10,000 synchronized L3-larval-stage progeny following exposure to 5 mM sodium arsenite for 30 min, which induced two- to threefold increases in *gcs-1* and *gst-7* expression and 6.6-, 10.6- and 5.5-fold increases, respectively, in *gst-4*, *dhs-8* and *sdz-8* mRNA levels [8]. RNA extraction was carried out using Trizol (Sigma) and *gcs-1*, *gst-7*, *gst-4*, *dhs-8*, *sdz-8* and *act-1* mRNA levels were determined using Superscript III Platinum SYBR Green One-Step qRT-PCR kit (Invitrogen, Paisley, UK) and Corbett Life Science Rotor-Gene 6000 system. mRNA levels were determined from a minimum of three replicate samples relative to *act-1* mRNA (For primer sequences see Additional file 1: Table S6). These were then compared with the levels in vector-control-treated animals. Experiments were repeated at least three times and the statistical significance of differences between *gcs-1*, *gst-7*, *gst-4*, *dhs-8* or *sdz-8* mRNA levels in RNAi-treated and control animals determined (Student's *T* test).

## Additional files

Additional file 1: Table S1. Genes required for growth, development or reproduction in the absence of *prdx‐2*. **Table S2.** RNAi clones that increased intestinal *gcs-1p::gfp* expression in *prdx-2* mutant. There were 50 clones that resulted in high levels of nuclear GFP throughout the intestine of at least 50% of the RNAi-treated *prdx-2 gcs-1p::gfp* worms (as illustrated in Figure 1). **Table S3.** RNAi clones that reduced intestinal *gcs-1p::gfp* expression in genome-wide RNAi screen in *prdx-2* mutants and their effect on the expression of a non-phase 2 intestinally expressed gene reporter *F09E5.3::gfp* [16] and a second phase 2 reporter gene *gst- 4p::gfp* [21] in wild-type (N2) animals under normal growth conditions*.* **Table S4.** Overlap between genes that are required for expression of *gcs-1p::gfp* in the *prdx-2* mutant (Table S3) and those identified by published RNAi screens of the same RNAi library for different phenotypes. **Table S5.** RNAi clones that prevented arsenite-induced intestinal *gcs-1p::gfp* expression in wild-type animals. Of the 90 RNAi clones that reduced intestinal *gcs-1p::gfp* expression in *prdx-2* mutant animals under normal growth conditions on each of four occasions (Table S3), 53 also prevented an increase in intestinal *gcs-1p::gfp* expression in wild-type (N2 *gcs-1p::gfp*) animals treated for 90 min with 1 mM arsenite. **Table S6.** Primer sequences used in quantitative PCR reactions. Additional file 2: Figure S1. Functional analysis of genes for which RNAi produced a synthetic lethal phenotype with *prdx-2* (Additional file 1: Table S1). **Figure S2.** Genes required for intestinal *gcs-1p::gfp* expression in *prdx-2* mutants represent a broad range of functional groups. **Figure S3.** The role of candidate genes identified by the screen in the regulation of *gst-4p*::*gfp* expression in *prdx-2* mutant animals. **Figure S4.** The effect of selected candidate RNAi (Table 1 and Figure 2B,C) on the arsenite resistance of wild-type animals. **Figure S5.** Effect of *csn-2*, *csn-4* and *csn-5* RNAi on intestinal expression of *gst-4p::gfp* in wild-type animals under normal growth conditions. **Figure S6.** UFD-2 is not required for arsenite-induced PMK-1 phosphorylation.

## Abbreviations

CnC: Cap and Collar
CSN: Cop9 signalosome
GCS: γ-glutamylcysteine synthetase
GFP: green fluorescent protein
GST: glutathione *S*-transferase
IPTG: isopropyl β-D-1-thiogalactopyranoside
IR: ionizing radiation
LB: Luria Broth
MAPK: mitogen-activated protein kinase
MAPKKK: mitogen-activated protein kinase kinase kinase
Nrf2: nuclear factor (erythroid-derived 2)-like 2
PCR: polymerase chain reaction
RNAi: RNA interference
ROS: reactive oxygen species

## References

[1] Liebler DC, Guengerich FP (2005). Elucidating mechanisms of drug-induced toxicity. Nat Rev Drug Discov.

[2] Board PG, Menon D (1830). Glutathione transferases, regulators of cellular metabolism and physiology. Biochim Biophys Acta.

[3] McElwee JJ, Schuster E, Blanc E, Piper MD, Thomas JH, Patel DS, Selman C, Withers DJ, Thornton JM, Partridge L, Gems D (2007). Evolutionary conservation of regulated longevity assurance mechanisms. Genome Biol.

[4] Tullet JM, Hertweck M, An JH, Baker J, Hwang JY, Liu S, Oliveira RP, Baumeister R, Blackwell TK (2008). Direct inhibition of the longevity-promoting factor SKN-1 by insulin-like signaling in *C. elegans*. Cell.

[5] Jaramillo MC, Zhang DD (2013). The emerging role of the Nrf2-Keap1 signaling pathway in cancer. Genes Dev.

[6] Furfaro AL, Macay JR, Marengo B, Nitti M, Parodi A, Fenoglio D, Marinari UM, Pronzato MA, Domenicotti C, Traverso N (2012). Resistance of neuroblastoma GI-ME-N cell line to glutathione depletion involves Nrf2 and heme oxygenase-1. Free Radic Biol Med.

[7] Zhang DD (2010). The Nrf2-Keap1-ARE signaling pathway: the regulation and dual function of Nrf2 in cancer. Antioxid Redox Signal.

[8] Oliveira RP, Porter Abate J, Dilks K, Landis J, Ashraf J, Murphy CT, Blackwell TK (2009). Condition-adapted stress and longevity gene regulation by *Caenorhabditis elegans* SKN-1/Nrf. Aging Cell.

[9] McMahon M, Lamont DJ, Beattie KA, Hayes JD (2010). Keap1 perceives stress via three sensors for the endogenous signaling molecules nitric oxide, zinc, and alkenals. Proc Natl Acad Sci USA.

[10] An JH, Blackwell TK (2003). SKN-1 links *C. elegans* mesendodermal specification to a conserved oxidative stress response. Genes Dev.

[11] Inoue H, Hisamoto N, An JH, Oliveira RP, Nishida E, Blackwell TK, Matsumoto K (2005). The *C. elegans* p38 MAPK pathway regulates nuclear localization of the transcription factor SKN-1 in oxidative stress response. Genes Dev.

[12] An JH, Vranas K, Lucke M, Inoue H, Hisamoto N, Matsumoto K, Blackwell TK (2005). Regulation of the *Caenorhabditis elegans* oxidative stress defense protein SKN-1 by glycogen synthase kinase-3. Proc Natl Acad Sci USA.

[13] Choe KP, Przybysz AJ, Strange K (2009). The WD40 repeat protein WDR-23 functions with the CUL4/DDB1 ubiquitin ligase to regulate nuclear abundance and activity of SKN-1 in *Caenorhabditis elegans*. Mol Cell Biol.

[14] Olahova M, Taylor SR, Khazaipoul S, Wang J, Morgan BA, Matsumoto K, Blackwell TK, Veal EA (2008). A redox-sensitive peroxiredoxin that is important for longevity has tissue- and stress-specific roles in stress resistance. Proc Natl Acad Sci USA.

[15] **WormBase.** [www.wormbase.org]

[16] Hunt-Newbury R, Viveiros R, Johnsen R, Mah A, Anastas D, Fang L, Halfnight E, Lee D, Lin J, Lorch A, McKay S, Okada HM, Pan J, Schulz AK, Tu D, Wong K, Zhao Z, Alexeyenko A, Burglin T, Sonnhammer E, Schnabel R, Jones SJ, Marra MA, Baillie DL, Moerman DG (2007). High-throughput *in vivo* analysis of gene expression in *Caenorhabditis elegans*. PLoS Biol.

[17] Wang J, Robida-Stubbs S, Tullet JM, Rual JF, Vidal M, Blackwell TK (2010). RNAi screening implicates a SKN-1-dependent transcriptional response in stress resistance and longevity deriving from translation inhibition. PLoS Genet.

[18] van Haaften G, Romeijn R, Pothof J, Koole W, Mullenders LH, Pastink A, Plasterk RH, Tijsterman M (2006). Identification of conserved pathways of DNA-damage response and radiation protection by genome-wide RNAi. Curr Biol.

[19] Samuelson AV, Carr CE, Ruvkun G (2007). Gene activities that mediate increased life span of *C. elegans* insulin-like signaling mutants. Genes Dev.

[20] Liao VH, Yu CW (2005). *Caenorhabditis elegans* gcs-1 confers resistance to arsenic-induced oxidative stress. Biometals.

[21] Leiers B, Kampkotter A, Grevelding CG, Link CD, Johnson TE, Henkle-Duhrsen K (2003). A stress-responsive glutathione S-transferase confers resistance to oxidative stress in *Caenorhabditis elegans*. Free Radic Biol Med.

[22] Simmer F, Moorman C, van der Linden AM, Kuijk E, van den Berghe PV, Kamath RS, Fraser AG, Ahringer J, Plasterk RH (2003). Genome-wide RNAi of *C. elegans* using the hypersensitive rrf-3 strain reveals novel gene functions. PLoS Biol.

[23] Merlet J, Burger J, Gomes JE, Pintard L (2009). Regulation of cullin-RING E3 ubiquitin-ligases by neddylation and dimerization. Cell Mol Life Sci.

[24] Kim DH, Feinbaum R, Alloing G, Emerson FE, Garsin DA, Inoue H, Tanaka-Hino M, Hisamoto N, Matsumoto K, Tan MW, Ausubel FM (2002). A conserved p38 MAP kinase pathway in *Caenorhabditis elegans* innate immunity. Science.

[25] Liberati NT, Fitzgerald KA, Kim DH, Feinbaum R, Golenbock DT, Ausubel FM (2004). Requirement for a conserved Toll/interleukin-1 resistance domain protein in the *Caenorhabditis elegans* immune response. Proc Natl Acad Sci USA.

[26] Leung CK, Hasegawa K, Wang Y, Deonarine A, Tang L, Miwa J, Choe KP (2014). A direct interaction between the WD40 repeat protein WDR-23 and SKN-1/Nrf inhibits binding to target DNA. Mol Cell Biol.

[27] Chen D, Wilkinson CR, Watt S, Penkett CJ, Toone WM, Jones N, Bahler J (2008). Multiple pathways differentially regulate global oxidative stress responses in fission yeast. Mol Biol Cell.

[28] Wakabayashi N, Itoh K, Wakabayashi J, Motohashi H, Noda S, Takahashi S, Imakado S, Kotsuji T, Otsuka F, Roop DR, Harada T, Engel JD, Yamamoto M (2003). Keap1-null mutation leads to postnatal lethality due to constitutive Nrf2 activation. Nat Genet.

[29] van der Hoeven R, McCallum KC, Cruz MR, Garsin DA (2011). Ce-Duox1/BLI-3 generated reactive oxygen species trigger protective SKN-1 activity via p38 MAPK signaling during infection in *C. elegans*. PLoS Pathog.

[30] Papp D, Csermely P, Soti C (2012). A role for SKN-1/Nrf in pathogen resistance and immunosenescence in *Caenorhabditis elegans*. PLoS Pathog.

[31] Nystrom T, Yang J, Molin M (2012). Peroxiredoxins, gerontogenes linking aging to genome instability and cancer. Genes Dev.

[32] Wu H, Pomeroy SL, Ferreira M, Teider N, Mariani J, Nakayama KI, Hatakeyama S, Tron VA, Saltibus LF, Spyracopoulos L, Leng RP (2011). UBE4B promotes Hdm2-mediated degradation of the tumor suppressor p53. Nat Med.

[33] Brenner S (1974). The genetics of *Caenorhabditis elegans*. Genetics.

[34] Kamath RS, Fraser AG, Dong Y, Poulin G, Durbin R, Gotta M, Kanapin A, Le Bot N, Moreno S, Sohrmann M, Welchman DP, Zipperlen P, Ahringer J (2003). Systematic functional analysis of the *Caenorhabditis elegans* genome using RNAi. Nature.

[35] **Stat Trek.** [http://stattrek.com/Tables/Hypergeometric.aspx]

