# Supplementary material for: Genome-wide screening identifies new genes required for stress-induced phase 2 detoxification gene expression in animals
Source: BMC Biol. 2014 Aug 14;12:64. doi: 10.1186/s12915-014-0064-6 (PMC4160539; doi:10.1186/s12915-014-0064-6)
Supplement: Additional file 1: Table S1. — Genes required for growth, development or reproduction in the absence of prdx‐2. Table S2. RNAi clones that increased intestinal gcs-1p::gfp expression in prdx-2 mutant. There were 50 clones that resulted in high levels of nuclear GFP throughout the intestine of at least 50% of the RNAi-treated prdx-2 gcs-1p::gfp worms (as illustrated in Figure 1). Table S3. RNAi clones that reduced intestinal gcs-1p::gfp expression in genome-wide RNAi screen in prdx-2 mutants and their effect on the expression of a non-phase 2 intestinally expressed gene reporter F09E5.3::gfp [16] and a second phase 2 reporter gene gst- 4p::gfp [21] in wild-type (N2) animals under normal growth conditions. Table S4. Overlap between genes that are required for expression of gcs-1p::gfp in the prdx-2 mutant (Table S3) and those identified by published RNAi screens of the same RNAi library for different phenotypes. Table S5. RNAi clones that prevented arsenite-induced intestinal gcs-1p::gfp expression in wild-type animals. Of the 90 RNAi clones that reduced intestinal gcs-1p::gfp expression in prdx-2 mutant animals under normal growth conditions on each of four occasions (Table S3), 53 also prevented an increase in intestinal gcs-1p::gfp expression in wild-type (N2 gcs-1p::gfp) animals treated for 90 min with 1 mM arsenite. Table S6. Primer sequences used in quantitative PCR reactions. [file 12915_2014_64_MOESM1_ESM.pdf]

Table S1 Genes required for growth, development or reproduction in the absence of *prdx-2*. 70 RNAi clones that resulted in lethal, sterile or reduced brood size phenotypes in the *prdx-2* mutant but had not previously been reported to result in these phenotypes in wild-type animals [1,2].

RNAi clone	Phenotype	function	Gene name
Y54G11A.5	sterile and lethal	stress defences	*ctl-2*, catalase
Y54G11A.6	sterile and lethal	stress defences	*ctl-1*, catalase
R07E5.2	sterile and lethal	stress defences	*prdx-3*, peroxiredoxin-3
W01A11.4	sterile and lethal	stress defences	*lec-10*, galectin
K04A8.8	sterile and lethal	stress defences	*spp-20*, saposin-like family
T07E3.4	sterile and lethal	signaling	F-box containing protein
Y71D11A.b	sterile and lethal	signaling	*fbxa-78*, F box A protein
F44D12.6	sterile and lethal	unknown function	
Y60A9.1	lethal	unknown function	
C47E8.3	lethal	unknown function	
K12B6.2	lethal	unknown function	uncharacterised conserved protein
F41E7.8	sterile	Stress defences	*cnc-8* caenacin peptide
H15N14.1	sterile	RNA processing	*adr-1*, tRNA-specific adenosine deaminase
F36A2.1	sterile	RNA processing	*cids-2*, contains a CTD of RNA Pol II-interacting domain
F13H8.2	sterile	RNA processing	subunit of 18S rRNA processing complex
F14B4.2	sterile	metabolism	hexokinase
W04G3.5	sterile	metabolism	phosphoribosylpyrophosphate synthetase-associated protein
C54D2.4	sterile	metabolism	*sul-3*, orthologous to the human gene ARYLSULFATASE B
Y110A2AL.12	sterile	membrane proteins	uncharacterised membrane protein
C49D10.3	sterile	membrane proteins	*srh-250*, serpentine receptor class H
Y57A10C.5	sterile	membrane proteins	*sre-25*, serpentine receptor class E
D1009.2	sterile	other	*cyn-8*, peptidyl-propyl cis-trans isomerase (cyclophilin family)
C36B7.7	sterile	signaling	*hen-1*, secreted protein with LDL receptor motif A
T20F7.3	sterile	signaling	Inositol polyphosphate multikinase
R03G5.5	sterile	unknown function	
F08F1.4	sterile	unknown function	
F47B10.4	sterile	unknown function	
Y62H9A.3	sterile	unknown function	unnamed protein
H38K22.4	sterile	unknown function	
Y110A2AM.1	sterile	unknown function	
Y17G7B.19	sterile	unknown function	
C17G10.7	sterile	unknown function	unnamed gene
C02B4.3	sterile	unknown function	
Y110A2AL.9	embryonic lethal	unknown function	
F46G10.1	embryonic lethal	unknown function	
F18G5.1	embryonic lethal	unknown function	
F08G2.7	embryonic lethal	unknown function	unnamed gene
Y46G5.x	embryonic lethal	metabolism	*acl-7*, Acyl-CoA:DHA-phosphate acyltransferase (DHAPAT)
C05D11.13	embryonic lethal	gene expression	Zn-finger protein and coiled-coil protein
F57G9.2	embryonic lethal	membrane proteins	*sre-30*, serpentine receptor class E
T26A5.1	embryonic lethal	membrane proteins	*wht-6*, ABC transporter
T28C12.3	sick	signaling	*fbxa-202*, f-box containing protein
K07G5.3	reduced brood size	signaling	C2 Ca^{2+}-binding motif-containing protein
F41D9.1	reduced brood size	signaling	*tbc-18* (Tre-2/Bub2/Cdc16 domain family) predicted GAP
C29F7.4	reduced brood size	gene expression	*fkh-3*, forkhead transcription factor
Y51H1A.5	reduced brood size	gene expression	*hda-6*, class II histone deacetylase
F56D2.4	reduced brood size	other	*uev-2*, ubiquitin-conjugating enzyme (UBC or E2) variant
F37B1.7	reduced brood size	Stress defences	*gst-18*, glutathione S transferase
F35D11.8	reduced brood size	Stress defences	*clec-137*, c-type lectin

C49D10.2	reduced brood size	gene expression	*nhr-166*, nuclear hormone receptor
F47C10.1	reduced brood size	gene expression	*nhr-185*, nuclear hormone receptor
Y102E9.1	reduced brood size	membrane proteins	*odr-4*, transmembrane protein needed for odorant response
R11G10.1	reduced brood size	membrane proteins	*avr-15*, glutamate-gated chloride channel subunit homologs
Y97E10A_263.b	reduced brood size	membrane proteins	*ncx-10*, putative Na[+]/Ca[2+] exchanger
F44F4.6	reduced brood size	metabolism	*gly-1*, transmembrane glycosyltransferase
F47G4.7	reduced brood size	metabolism	*smd-1*, S-adenosylmethionine decarboxylase
Y102A5C.1	reduced brood size	signaling	*fbxa-206*, protein containing an F box
F56C9.10	reduced brood size	signaling	WD40 repeat containing protein
C35E7.9	reduced brood size	unknown function	uncharacterized, contains major sperm protein(MSP) domain
F53E10.1	reduced brood size	unknown function	uncharacterised Fe-S protein
T08A9.5	reduced brood size	unknown function	
R06C7.6	reduced brood size	unknown function	uncharacterised conserved protein
F54D10.7	reduced brood size	unknown function	
B0284.2	reduced brood size	unknown function	uncharacterised protein
F49C5.7	reduced brood size	unknown function	
F38H4.3	reduced brood size	unknown function	
Y57G11A.2	reduced brood size	unknown function	uncharacterised protein
F02C9.2	reduced brood size	unknown function	uncharacterised conserved protein
F32D8.8	reduced brood size	unknown function	
T22E5.1	reduced brood size	unknown function	

1. Kamath RS, Fraser AG, Dong Y, Poulin G, Durbin R, et al. (2003) Systematic functional analysis of the Caenorhabditis elegans genome using RNAi. Nature 421: 231-237.

2. Rual JF, Ceron J, Koreth J, Hao T, Nicot AS, et al. (2004) Toward improving Caenorhabditis elegans phenome mapping with an ORFeome-based RNAi library. Genome Res 14: 2162-2168.

Table S2. RNAi clones that increased intestinal *gcs-1p::gfp* expression in *prdx-2* mutant 50 clones which resulted in high levels of nuclear GFP throughout the intestine (as illustrated in Fig.1) of at least 50% of the RNAi-treated *prdx-2 gcs-1p::gfp* worms are listed. Description of the predicted function and potential human homolog of each gene, are shown. 'nematodes only' indicates that no homologs identified outside *Caenorhabditis briggsae*, *Caenorhabditis brenneri*, *Caenorhabditis remanei*, *Caenorhabditis japonica* or *Pristionchus pacificus*.

**RNAi clone**	**Human Homolog**	**Description**
T12F5.4	none	putative transcription factor ASH1/LIN-59
T03F1.1	UBA5	molybdopterin biosynthesis-related protein
T03F1.8	GUK1	guanylate kinase
E01A2.i	GCLM	glutamate-cysteine ligase regulatory subunit
D2030.9	DCAF11	*wdr-23*, WD40 repeat containing protein
T28B8.5	SCNN1A	*del-4*, Non voltage-gated ion channels (DEG/ENaC family)
T19A6.3	TMEM188	uncharacterised conserved protein
Y106G6H.10	none	
C34B2.4	LMO4	adaptor protein Enigma and related PDZ-LIM proteins
Y47H9C.12	none	uncharacterised protein
W02D9.3	HMG20B, HMG20A	HMG-box containing protein
T06G6.9	VBP1	*pfd-3,* putative molecular chaperone prefoldin
F44F1.6	nematodes only	uncharacterised coiled-coil containing protein
T02H6.8	none	unnamed protein
Y50F7A.1	RAP1GAP2	merged into F53A10.2, Rap1GAP homolog
F59H6.3	none	unnamed protein
C08B11.6	ACTR6	actin-related protein
M176.2	GSS	glutathione synthetase
ZC47.13	nematodes only	*fbxa-50*, contains an F-box and FTH/DUF38 motif, which may mediate protein-protein interactions
C38D4.7	nematodes only	
F10E9.1	nematodes only	
ZC262.4	none	
R05D3.11	SETDB2	*met-2,* histone methyltransferase
F54F2.2	MLLT10	*zfp-1,* leucine zipper, zinc finger, and PHD/LAP domain-containing protein
K03H1.5	SUSD2	mucin/alpha tectorin
Y53G8A_9248.c	NDUFA9	*Y53G8AL.2*, NADH:ubiquinone oxidoreductase
T20D3.1	nematodes only	*clec-183*, c-type lectin
F36H1.4	nematodes only	*lin-3*, member of the EGF family of peptide growth factors
B0035.11	LEO1	uncharacterised conserved protein

C26C9.2	none	Merged into T06A10.2
F26D10.1	CD69, CLEC4A, CLEC5A, CLEC4D, CLEC6A	*clec-87* putatively secreted C-type lectin, paralogous to CLEC-88, C25B8.4, F26D10.12, ZK858.3
F26D10.2	ACACA	T28F3.5
Y37E11A_94.e	nematodes only	Y37E11AL.6 predicted secreted protein, two cadherin domains and a C-terminal EGF-like domain
Y9C9A_54.a	SQRDL	targets Y9C9A.16, 21ur-2020, 21ur-11733, 21ur-9201
F49F1.1	none	secreted surface protein
R08C7.1	none	
H24K24.a	ADH5	targets H24K24.3 alcohol dehydrogenase class III and Y50D4C.2 alcohol dehydrogenase type III
ZK697.6	GSTA3 and others	*gst-21*, glutathione s transferase
ZK697.11	nematodes only	*srh-199*, serpentine receptor class H
W02H5.a	DAK	W02H5.8, Dihydroxyacetone kinase/glycerone kinase
C46A5.6	nematodes only	
C55A1.10	none	*srh-168*, serpentine receptor class H
F54B8.5	nematodes only	*abf-3,* may play a role in innate immunity from sequence similarity
F53B1.4	TGDS	putative NAD+-dependent epimerases
H03G16.4	nematodes only	*fbxa-96*, f box A protein
H03G16.5	none	
K09E9.1	none	synaptic vesicle transporter SVOP and related transporters (major facilitator superfamily)
T27A8.3	METAP2	metallopeptidase
Y13C8A.a	none	targets F46C3.7 and Y13C8A.1, uncharacterised protein
B0302.4	none	

Table S3. RNAi clones which reduced intestinal *gcs-1p::gfp* expression in genome-wide RNAi screen in *prdx-2* mutant and their effect on the expression of a non-phase 2 intestinally expressed gene reporter *F09E5.3::gfp* and a second phase 2 reporter gene *gst-4p::gfp* in wild-type (N2) animals under normal growth conditions. Each RNAi clone reduced intestinal *gcs-1p::gfp* to undetectable levels (Fig.1) in at least 50% of the RNAi-treated animals but none were observed to prevent pharyngeal expression of *gcs-1p::gfp*. The 355 RNAi clones are listed according to the number of times they reduced *gcs-1p::gfp* out of the 4 times in which they were screened. RNAi clones which affected intestinal *F09E5.3::gfp* (33) or *gst-4p::gfp* (19) expression on at least 2 out of 3 occasions on which they were scored are indicated. Human homologs are indicated where known. Gene descriptions were derived from WormBase.

RNAi clone	Reduced *gcs-1p::gfp*	Effect on *F09E5.3::gfp*	Effect on *gst4p::gfp*	Human homolog	Gene description
C26C6.1	4/4	decreased	decreased	PBRM1	*pbrm-1*, interacts with two or more components of the EGF/RAS signalling pathway
R134.2	4/4	-	-	GUCY/NPR1/2	*gcy-2*, natriuretic peptide receptor predicted to function in chemosensory signal transduction
AH6.1	4/4	-	-	GUCY/NPR1/2	*gcy-1*, natriuretic peptide receptor, guanylate cyclase
K04D7.1	4/4	decreased	decreased	GNB2L	*rack-1*, G protein beta subunit-like protein
F13B10.1	4/4	-	-	SARM1	*tir-1*, TIR (Toll and Interleukin 1 Receptor) domain protein
R12B2.1	4/4	-	-	SMAD	*sma-4*, TGFbeta receptor signaling protein SMAD
C06E8.1	4/4	decreased	decreased	GLRA	targets F11H8.2 (*lgc-38*), an UNC-49-like GABA receptor subunit
F57F5.5	4/4	-	-	PRKC, RBPMS	sequencing indicates clone may target multiple genes including *mec-8* and *pkc-1*
F47A4.2	4/4	-	-	MED12L, MED12	*dpy-22*, homologous to the transcriptional mediator protein TRAP230, which is involved in WNT and RAS signaling
C16A3.8	4/4	-	-	THOC2	*thoc-2*, THO complex (transcription factor/nuclear export) subunit
C28H8.3	4/4	-	-	Q96ND6	predicted helicase, DEAD-box superfamily
W01B6.9	4/4	-	-	COG-7	*ndc-80*, Centromere-associated protein HEC1
D2096.8	4/4	decreased	-	NAP1L	related to NAP (Nucleosome Assembly Protein) family of proteins involved in chromatin remodeling
T26A8.4	4/4	-	-	ZC3H4/6	component of the conserved Ccr4-Not deadenylase complex that regulates transcription and RNA degradation
C17G10.1	4/4	-	-	OGFOD1	Predicted component of NuA3 histone acetyltransferase complex
B0286.4	4/4	decreased	decreased	CNOT2	*ntl-2*, predicted transcriptional regulator
E01A2.f	4/4	-	-	NKAPL, NKAP	E01A2.4 NFKB activating protein homolog
B0025.2	4/4	-	increased	COPS2	*csn-2,* COP9 signalosome subunit CSN2
T05H10.5	4/4	-	decreased	UBE4A/UBE4B	*ufd-2*, ubiquitin fusion degradation protein-2
Y102A5A.1	4/4	-	-	CAND1/CAND2	*cand-1*, TATA-binding protein-interacting protein
F08B12.2	4/4	-	-	PEX12	*prx-12*, Predicted E3 ubiquitin ligase involved in peroxisome organization
F27D4.1	4/4	-	-	ETFA	electron transfer flavoprotein
Y49E10.4	4/4	-	-	PDIA6	thioredoxin/protein disulphide isomerase
D2023.1	4/4	decreased	-	CYPB5A/CYPB5B	cytochrome B5
K05D4.4	4/4	decreased	-	several eg CYP2A6	*cyp-33D1*, Cytochrome P450 CYP2 subfamily
M60.1	4/4	-	-	PP11	targets M60.2, placental protein 11
D2013.10	4/4	-	-	TMEM41B	*tag-175*, functionally uncharacterised, predicted membrane protein
B0416.5	4/4	-	-	MFSD7	permease of the major facilitator superfamily
F20H11.3	4/4	-	-	MDH2	*mdh-1*, homolog of malate dehydrogenase that is predicted to be mitochondrial
C34F11.3	4/4	-	-	AMPD1	adenosine monophosphate deaminase
C06A8.1	4/4	-	-	MTHFR	orthologous to human MSH HOMEOBOX HOMOLOG 1 (MTHFR) mutated in homocystinuria

F32B6.2	4/4	-	-	MCCC1	ortholog of human ALPHA METHYLCROTONOYL-COENZYME A CARBOXYLASE 1
T12A2.1	4/4	-	-	AMDHD1	atrazine chlorohydrolase/guanine deaminase
F41H10.7	4/4	-	-	ELOVL3/6	*elo-5*, polyunsaturated fatty acid (PUFA) elongase
Y57A10C.6	4/4	decreased	decreased	SCP2	*daf-22*, catalyzes the final step in peroxisomal fatty acid beta-oxidation
F21H12.4	4/4	decreased	decreased	PTCH2	*ptc-2*, sterol-sensing domain protein
E04F6.3	4/4	-	-	HSD17B4	*maoc-1*, peroxisomal multifunctional beta-oxidation protein
C44B11.3	4/4	-	-	TUBA1/TUBA4	*mec-12*, alpha-tubulin required for normal mechanosensory response to gentle touch
ZK617.1	4/4	-	-	OBSL1, OBSCN	*unc-22* encodes twitchin
D1046.1	4/4	-	-	CPSF6	*cfim-2*, mRNA cleavage factor I subunit/CPSF subunit
F32B6.3	4/4	decreased	-	PRPF18	U5 snRNP-associated RNA splicing factor
Y116A8C.32	4/4	-	-	SF1	*sfa-1*, Splicing factor 1/branch point binding protein (RRM superfamily)
K02F3.11	4/4	-	-	RNPS1	*rnp-5*, splicing factor
C16A3.3	4/4	-	-	PDCD11	*let-716*, rRNA processing protein Rrp5
R11A5.1	4/4	-	-	AP3B1/AP3B2	*apb-3*, adaptin predicted to be involved in formation of intracellular transport vesicles
Y119D3_445.b	4/4	-	-	PCDH	*cdh-12*, cadherin, predicted to function in cell adhesion
M01E11.7	4/4	-	-	TNS1/3/4, TENC1	*tag-163,* Focal adhesion protein Tensin
K04G7.12	4/4	-	-	SYF2	targets K04G7.11
C18F10.5	4/4	-	-	nematodes only	*srg-2*, serpentine receptor class G
Y111B2B.b	4/4	-	-	nematodes only	targets ZC482.2, predicted receptor. Secondary target Y69A2AR.11
C45H4.1	4/4	-	-	nematodes only	*srbc-16*, serpentine receptor class BC.
T27B7.1	4/4	-	-	nematodes only	*nhr-59*, nuclear hormone receptor
F46E10.11	4/4	-	-	nematodes only	*hpo-26,* fibrillins and related proteins containing Ca^{2+}-binding EGF-like domains
C47G2.1	4/4	-	-	nematodes only	*cut-1*, cuticulin precursor
T28H11.2	4/4	-	-	nematodes only	*srm-1*, serpentine receptor class m
C29E6.1	4/4	-	-	nematodes only	*let-653*, mucin-like protein similar to the highly glycosylated mucins secreted by the apical surface of epithelia
M01G12.4	4/4	-	-	nematodes only	*srw-111*, serpentine receptor class W
T06G6.1	4/4	-	-	nematodes only	*sra-23*, serpentine receptor, class a
T10B9.1	4/4	-	-	nematodes only	*cyp-13A4*, Cytochrome P450 CYP3/CYP5/CYP6/CYP9 subfamilies
T18H9.1	4/4	-	decreased	nematodes only	*grd-6* contains a C-terminal Ground domain
C35C5.1	4/4	-	decreased		*sdc-2*, represses transcription of X chromosomes to achieve dosage compensation
Y105C5A.n	4/4	-	-	nematodes only	targets M199.8 and Y105C5A.26
F22F7.4	4/4	-	-	nematodes only	extracellular protein with conserved cysteines
T12A2.5	4/4	-	-	nematodes only	
F46F11.6	4/4	decreased	-	nematodes only	
W01A8.5	4/4	-	-	nematodes only	
F59A6.2	4/4	-	-	nematodes only	
R148.2	4/4	-	-	nematodes only	
B0244.4	4/4	-	-	nematodes only	
Y75B8A.33	4/4	-	-	nematodes only	
T12D8.5	4/4	-	-	nematodes only	
F21F8.5	4/4	-	-	nematodes only	
F25B3.4	4/4	-	-	nematodes only	

R13H4.7	4/4	-	-	nematodes only	
F08G12.5	4/4	-	-	nematodes only	
D1053.4	4/4	-	-	nematodes only	
F54C1.3	4/4	decreased	-		*mes-3*, member of polycomb-like chromatin repressive complex
C05D11.1	4/4	-	-		predicted Zn2+-dependent endopeptidase, insulinase superfamily
ZK792.3	4/4	-	-		*inx-9* encodes an integral transmembrane channel protein that is a structural component of invertebrate gap junctions
F58B6.1	4/4	-	-		
T26A5.2	4/4	-	-		
T19B10.2	4/4	-	-		
C44B9.2	4/4	-	-		
Y111B2C.a	4/4	-	-		targets Y111B2A.4, and Y41D4B.15
C18F10.2	4/4	-	-		uncharacterized protein, contains major sperm protein (MSP) domain
C24A11.2	4/4	-	-		
W02D9.8	4/4	-	-		
F49E12.7	4/4	-	-		
C50E3.8	4/4	-	-		
F35B3.3	4/4	-	-		
F07C3.4	3/4	-	-	RCBTB1/2	*glo-4*, guanine nucleotide exchange factor required for biogenesis of the lysosome-related gut granules
F31F4.5	3/4	-	-	several	targets Y4C6A.1, protein tyrosine kinase
W09C5.7	3/4	-	-	SACM1L	phosphoinositide phosphatase SAC1
T13F2.3	3/4	-	-	PAXIP1	*pis-1,* ortholog of mammalian Pax transcription activation domain interacting protein PTIP
T16H12.4	3/4	-	decreased	GTF2H2	RNA polymerase II transcription initiation/nucleotide excision repair factor TFIIH, subunit SSL1
H22K11.1	3/4	decreased	-	CTSD, NAPSA, REN,PGC	*asp-3*, aspartyl protease , also homologous to CTSE
C26B2.6	3/4	-	-	ELP4	*elpc-4*, RNA polymerase II elongator complex protein component
C28H8.11	3/4	-	-	TDO2	tryptophan 2,3-dioxygenase
T23B12.7	3/4	-	-	DNAJC17	*dnj-22*, Molecular chaperone (DnaJ superfamily)
F52B5.1	3/4	-	-	SLC4A9	*abts-1*, Na+-independent Cl/HCO3 exchanger AE1 and related transporters
F36H1.2	3/4	-	-	NKPD1	*tag-144*, Integral membrane ankyrin-repeat protein Kidins220 (protein kinase D substrate)
F35G12.7	3/4	-	-	CUTA	
F59A3.1	3/4	-	-	CPM, CPD	encodes one of two C. elegans carboxypeptidase D homologs
Y41D4A_3192.a	3/4	-	-	QRSL1	targets Y41D4A.6, amidases
K07C11.4	3/4	-	-	several	carboxylesterase and related proteins
T23B12.4	3/4	-	-	NAA35	Glucose-repressible protein and related proteins
W03G11.1	3/4	-	-	MARCO	*col-181*, collagen
T26A5.9	3/4	-	-	DYNLL2/DYNLL1	*dlc-1*, Dynein light chain type 1
T08G11.1	3/4	-	-	VPS13A/B/C	vacuolar protein sorting-associated protein
W06A7.3	3/4	-	-	RTN4	*ret-1*, reticulon protein
F44A2.5	3/4	-	-	KIAA0427-001	MIF4G domain-containing protein
F36D4.5	3/4	-	-	C1orf121	uncharacterized conserved protein
T19A5.1	3/4	-	-	TTC17	uncharacterized conserved protein, contains TPR repeats
F33H1.3	3/4	decreased	-	WBP11	uncharacterised conserved low complexity protein
C48B6.8	3/4	-	-	BBS9	*bbs-9*, bardet beidl syndrome protein. Predicted coiled-coil domain.

R09E12.7	3/4	-	-	nematodes only	targets R09E12.4 and *str-238*, 7TM receptor
F48G7.1	3/4	-	-	nematodes only	*srj-37*, 7TM receptor
C45H4.15	3/4	-	-	nematodes only	*str-255*, 7TM receptor
C45H4.6	3/4	-	-	nematodes only	*srbc-18*, serpentine receptor class BC like
C45H4.11	3/4	-	-	nematodes only	*srbc-20*, serpentine receptor class BC like
C45H4.12	3/4	-	-	nematodes only	*str-52*, serpentine receptor
C34C6.1	3/4	-	-	nematodes only	*srg-15*, serpentine receptor class g.
F32B6.1	3/4	-	-	nematodes only	*nhr-4*, nuclear hormone receptor
F44C8.11	3/4	-	-	nematodes only	*nhr-96*, nuclear hormone receptor
F36F12.6	3/4	-	-	nematodes only	*clec-208*, c type lectin
C03A7.14	3/4	-	-	nematodes only	*abu-8,* transmembrane protein with multiple cysteine-rich repeats; is induced by blockage of the UPR
Y105C5A.d	3/4	-	-	nematodes only	targets many pqn proteins; *pqn-90*, *pqn-78*, *pqn-91*, *pqn-76*, *pqn-79* and *abu-5*.
Y105C5A.c	3/4	-	-	nematodes only	targets many genes including *pqn-76*, *pqn-79*, *abu-5* (activated in unfolded protein response)
T01H10.5	3/4	-	-	nematodes only	*lgc-16*, ligand gated ion channel acetylcholine receptor
F59F3.2	3/4	-	-	nematodes only	*col-177*, collagen
ZC47.13	3/4	-	-	nematodes only	*fbxa-50*, contains an F-box, predicted to mediate protein-protein interactions
Y49F6C.3	3/4	-	-	nematodes only	*bath-9*, Uncharacterized protein, contains BTB/POZ domain
F23F12.4	3/4	-	-	nematodes only	*sdz-15*, skn-1 dependent zygotic transcript. Contains an F box
F22F7.3	3/4	-	-	nematodes only	extracellular protein with conserved cysteines
R11G11.6	3/4	-	-	nematodes only	predicted alpha-helical protein
F09F7.5	3/4	-	-	nematodes only	encodes 3 isoforms of an unfamiliar protein required for brood size, growth, and for normally short lifespan
M7.10	3/4	decreased	-	nematodes only	predicted alpha helical protein
C29F9.1	3/4	-	-	nematodes only	uncharacterised protein
Y57G11B.1	3/4	-	-	nematodes only	uncharacterised protein
Y105C5A.m	3/4	-	-	nematodes only	targets Y105C5A.14, and W05H12.2
T28D9.1	3/4	-	-	nematodes only	
F44G4.7	3/4	-	-	nematodes only	
M176.8	3/4	-	-	nematodes only	
M01G12.6	3/4	-	-	nematodes only	
T21E8.5	3/4	-	-	nematodes only	
F54B8.1	3/4	decreased	-	nematodes only	
F45F2.11	3/4	-	-	nematodes only	
Y105C5A.i	3/4	-	-		targets Y105C5A.10, predicted transposase
Y69H2.4	3/4	-	-		superseded gene name
T16A9.1	3/4	-	-		transposon
C06B8.5	3/4	-	-		transposon
R09E12.6	3/4	-	-		transposon
Y82E9BR.22	3/4	-	-		
Y55F3A_747.a	3/4	-	-		targets Y55F3BR.10
Y69A2A_7278.m	3/4	-	-		no RNAi targets identified
Y69A2A_7278.d	3/4	-	-		target Y69A2AR.27
Y53C10A.10	3/4	decreased	-		

Y25C1A.6	3/4	-	decreased		
C05B10.2	3/4	-	-		merged into R13H7.2
Y39C12A.h	3/4	-	-		Y39C12A.9
Y51H4A.b	3/4	decreased	-		targets Y51H4A.2
F55C7.2	3/4	-	-		
F52D10.2	2/4	-		FAM43A/B	uncharacterized conserved protein, contains phosphotyrosine interaction (PI) domain
H09G03.2	2/4	-	-	FRMPD3	*frm-8,* FERM domain protein
M01A10.1	2/4	-	-	KHSRP, FUBP1/3	predicted to regulate gene expression through binding mRNA or ssDNA
F21D5.2	2/4	-	-	OTUD6B	OUT (ovarian tumour-like) cysteine protease
D2085.2	2/4	-	-	ATG10	*atg-10*, yeast atg homolog. Uncharacterised conserved protein.
C05D11.12	2/4	-	-	ETFDH	*let-721*, Electron transfer flavoprotein ubiquinone oxidoreductase
B0403.4	2/4	-	-	PDAI6	*tag-320*, Thioredoxin/protein disulfide isomerase
ZK520.3	2/4	-	-	WDR19	*dyf-2*, expression is regulated by DAF-19; *dyf-2* mutants are longer-lived and heat-stress resistant
C47E8.4	2/4	-	-	FAM50A/50B	encodes a conserved protein that is a member of the FAM50A/XAP5 family
F25G6.6	2/4	-	-	NARS2	*nars-2*, Asparagine synthase (glutamine-hydrolyzing)
ZC395.6	2/4	-	-	TRIT1	*gro-1*, tRNA delta(2)-isopentenylpyrophosphate transferase
C41C4.8	2/4	-	-	VCP	*cdc-48.2*, AAA+ type ATPase
C25A1.5	2/4	-	decreased	FA2H,	cytochrome B5, sphingolipid fatty acid hydroxylase
F01G10.3	2/4	-	-	EHHADH	*ech-9*, Hydroxyacyl-CoA dehydrogenase/enoyl-CoA hydratase
ZK973.g	2/4	-	-	SYNE1	*anc-1*, abnormal nuclear anchorage
ZK593.8	2/4	-	-	FICD	Huntingtin interacting protein HYPE
K12F2.1	2/4	-	-	MYH1/2/3/8/13/15	*myo-3*, Myosin class II heavy chain
F46C5.9	2/4	-	-	TBL2	WD40 repeat protein
Y51H7C_255.g	2/4	-	decreased	COG4	*cogc-4,* Golgi transport complex COD1 protein
T24B8.7	2/4	-	-	USP18/41/48/24/9X/9Y	
F35H10.6	2/4	-	-	UXT	
Y69H2.13	2/4	-	-	VWDE	*tag-336*
F41C6.5	2/4	-	-	MARCO	*col-173*, collagen
F08D12.14	2/4	-	-	ESRRA, ESR1	merged into K12H6.1, *nhr-119* nuclear hormone receptor
T09F5.1	2/4	-	-	B3GALT1	galactosyltransferases
F23C8.7	2/4	-	-	nematodes only	protein tyrosine kinase
Y53F4C.f	2/4	-	-	nematodes only	targets Y53F4B.25 (fibronectin), Y53F4B.8 and several 21U-RNAs
F58H7.8	2/4	-	-	nematodes only	*fbxc-3*, F-box domain
F23F12.3	2/4	-	-	nematodes only	Synaptic vesicle transporter SVOP and related transporters (major facilitator superfamily)
F48C1.7	2/4	-	-	nematodes only	*spe-11*, defective spermatogenesis
Y71G12A_201.b	2/4	-	-	nematodes only	*lin-65*, required for fertility
Y105C5A.j	2/4	-	-	nematodes only	targets *srg-19* and *srh-72*, serpentine receptors
T01G6.9	2/4	-	-	nematodes only	*str-256*, 7TM receptor
T20D3.1	2/4	-	-	nematodes only	*clec-183*, c-type lectin
Y69A2A_2991.c	2/4	-	-	nematodes only	targets Y69A2AR.19, protein tyrosine phosphatase contains WSN domain.
F58G4.4	2/4	-	-	nematodes only	*sdz-23*, skn-1 dependent zygotic transcript
C06E4.7	2/4	-	-	nematodes only	*glb-2*, globin related

C49A9.3	2/4	-	-	nematodes only	uncharacterised protein with ubiquitin fold
R05A10.6	2/4	-	-	nematodes only	extracellular protein with conserved cysteines
F36F12.1	2/4	-	-	nematodes only	extracellular protein with conserved cysteines
B0478.3	2/4	-	-	nematodes only	
C38D4.1	2/4	-	-	nematodes only	
K02E10.3	2/4	decreased	-	nematodes only	targets K02E10.4
H23L24.b	2/4	-	-	nematodes only	targets H23L24.4
T27E7.3	2/4	-	-	nematodes only	
F55B11.3	2/4	-	-	nematodes only	
Y57G11C.8	2/4	-	-	nematodes only	
C35D10.8	2/4	decreased	-	nematodes only	
Y26D4A.8	2/4	-	-	nematodes only	
C39H7.4	2/4	-	-	nematodes only	
T05A12.3	2/4	-	-	nematodes only	
C03B1.2	2/4	-	-	nematodes only	
F20C5.6	2/4	-	-	nematodes only	
F38E11.6	2/4	-	-	nematodes only	
F55B11.4	2/4	-	-	nematodes only	
Y57G11A.5	2/4	-	-	nematodes only	
F13E9.9	2/4	-	-	nematodes only	
T27E7.1	2/4	-	-	nematodes only	
H12I19.3	2/4	-	-		*srz-30*, serpentine receptor class Z
T27E7.2	2/4	-	-		*srh-65*, serpentine receptor class h
Y57G11C.27	2/4	-	-		*srt-51*, serpentine receptor class t
B0413.2	2/4	-	-		targets *tsp-13*, Tetraspanin family integral membrane protein
Y57G11A.3	2/4	-	-		*tag-273*
ZK262.5	2/4	-	-		transposon
C02F4.3	2/4	-	-		
K08D8.1	2/4	-	-		
Y41E3.5	2/4	-	-		
R12B2.6	2/4	-	-		
F21F8.6	2/4	-	-		
F14H3.6	2/4	-	-		
R03G8.3	2/4	-	-		
ZK643.3	1/4	-	-	CRHR2/1,	adenylate cyclase-coupled calcitonin receptor also orthologous to PTH2R/1R, CALCR, CALCRL
C01B10.3	1/4	-	-	INPP5A	inositol polyphosphate 5 phosphatase, type I
F01D4.3	1/4	-	-	FER	tyrosine kinase
Y105C5A.x	1/4	-	-	MOS, MAP3K7	targets Y105C5A.24, tyrosine kinase and several 21URNAs
F38E1.7	1/4	-	-	WNT10A/9A/9B/10B	*mom-2*, wnt family of developmental regulators
F47D12.4	1/4	-	-	HMGB1/2/3/4	*hmg-1.2*, HMG box-containing
ZC168.3	1/4	-	-	ORC5	origin recognition complex, subunit 5
ZK593.4	1/4	-	-	KDM5A/B/C/D, JARID2	*rbr-2*, retinoblastoma binding related. Histone H3 lysine 4
Y57G11C.25	1/4	-	-	ZFP36L2/1, ZFP36	*ccch-5,* CCCH-type Zn-finger protein transcription factor

C50F4.7	1/4	-	-	HIST1H4G/4H/4K/4A/4F	*his-37*, H4 histone
C34C6.8	1/4	-	-	VENTX	*ceh-7*, homeodomain transcription factor
F08D12.1	1/4	-	-	SRP72	signal recognition particle homolog
H06H21.3	1/4	-	decreased	EIF1AX, EIF1AY	translation initiation factor
F37B1.1	1/4	-	-	GSTA1/2/3/4/5	*gst-24,* glutathione-S-transferase
C02D5.3	1/4	-	-	GSTO2/1	*gsto-2*, glutathione S transferase
Y37A1C.1	1/4	-	-	SLC12A1/2/3	*nkcc-1*, Na-K-Cl cotransporter
C26H9A.1	1/4	-	-	ATP6V0A4/1/2, TCIRG1	*vha-7*, vacuolar H ATPase
T08G11.4	1/4	-	-	TGS1	methylase, negatively regulates *gpdh-1*
K12H6.3	1/4	-	-	FUT3/4/5/6/7/9	*fut-4,* fucosyl transferase
F49E12.9	1/4	-	-	C5orf4	C-4 sterol methyl oxidase
T09F3.3	1/4	-	-	GAPDHS, GAPDH	*gpd-1*, glyceraldehyde 3-dehydrogenase
Y46G5.kk	1/4	-	-	GYS1, GYS2	*gsy-1*, glycogen synthase 1
ZK688.3	1/4	-	-	FAHD2A/B, FAHD1	predicted fumarylacetoacetate hydralase
H04M03.1	1/4	-	-	PCK1/2	phosphoenolpyruvate carboxykinase
Y67D8A_369.a	1/4	-	-	PHKB	Y67D8A.1, phosphorylase kinase, and Y67D8A.9
JC8.4	1/4	-	-	ADAT2	cytosine deaminase
Y76B12C_67.c	1/4	-	-	OSBPL6, RP4-604K5.1,	Y76B12C.3, selenoprotein , also homologous to ATP1B1
Y40B10A.g	1/4	-	decreased	COMTD1	Y40B10A.2, O-methyltransferase
R05H10.2	1/4	-	decreased	RBM28	*rbm-28*, nucleolar protein fibrillarin NOP77
R07G3.3	1/4	-	decreased	TPR	*npp-1*, nuclear pore complex, p54 component
ZK973.i	1/4	-	-	SYNE2, SYNE1, CLMN	targets *anc-1*, which affects positioning of nuclei and mitochondria within the cytoskeleton, and other genes...
D2024.6	1/4	-	-	CAPZA2	*cap-1*, F-actin capping protein
F08B4.1	1/4	-	-	SAGE1, CT45A1, INTS6, DDX26B	*dic-1*, essential for normal mitochondria morphology and function
Y53C12A.4	1/4	-	-	CAB39L	*mop-25.2*, negatively regulates dynein
B0336.9	1/4	-	-	SFSWAP	*swp-1*, mRNA binding factor
R07E5.14	1/4	-	-	RBM8A	*rnp-4*, RNA binding protein, putative member of exon-exon junction complex
C17E4.5	1/4	-	-	PABPN1L	*pabp-2*, polyA binding protein, splicing factor
C44E4.4	1/4	-	-	SSB	RNA binding La
Y71G12A_201.a	1/4	-	-	DDX27	targets Y71G12B.8, DEAD box helicase, and Y71G12B.25
W07E6.1	1/4	-	-	NOP2	*nol-1*, tRNA and rRNA cytosine-C5-methylase (nucleolar protein NOL1/NOP2)
C06G3.10	1/4	-	-	COG2	*cogc-2*, low density lipoprotein receptor
Y48B6A.4	1/4	-	decreased	CHRFAM7A, CHRNA7	*eat-2*, acetylcholine receptor
C01F6.7	1/4	-	-	SLC9A3R1	*tag-60*, acetylcholine transmission
Y39B6B.a	1/4	-	-	SDAD1	*pro-3*, proximal proliferation in germline
Y49A3A.3	1/4	-	-	CCDC76	uncharacterised conserved protein
R12B2.2	1/4	-	-	BLZF1	
F37C12.1	1/4	-	-	CCDC94	uncharacterized conserved protein
C16C10.6	1/4	-	-	CCDC55	*ccdc-55*, uncharacterised conserved protein
D2085.5	1/4	-	decreased	RALGAPB	uncharacterised conserved protein, short lifespan, age associated fluorescence increased
W09H1.3	1/4	-	decreased	C3orf33	uncharacterised conserved protein
F46C8.6	1/4	-	-	COL17A1	*dpy-7*, collagen

M153.2	1/4	-	-	MFSD11	uncharacterised conserved protein
R148.1	1/4	-	-	nematodes only	*mks-1 / xbx-7*
F41D3.4	1/4	-	-	nematodes only	*oac-27*, integral membrane O-acetyltransferase
T06C10.2	1/4	-	-	nematodes only	*srt-73*, serpentine receptor class T
T12A2.13	1/4	-	-	nematodes only	*srg-6*, serpentine receptor class G
F10A3.13	1/4	-	-	nematodes only	*str-108*, serpentine receptor
F54B8.10	1/4	-	-	nematodes only	*srbc-52*, serpentine receptor, class BC
K03D3.4	1/4	-	-	nematodes only	*srz-61,* serpentine receptor class Z
C35D6.2	1/4	-	-	nematodes only	*srh-227*, serpentine receptor class H
F48G7.12	1/4	-	-	nematodes only	predicted small molecule kinase
F20D6.5	1/4	-	-	nematodes only	predicted small molecule kinase
H24O09.b	1/4	-	decreased	nematodes only	*fbxb-72*, F box B protein, predicted transposase
W02D7.6	1/4	-	-	nematodes only	C2H2-type Zn-finger
R09B5.3	1/4	-	-	nematodes only	*cnc-2*, caenacin, secreted antimicrobial peptide regulated by TGFbeta pathway
T06C12.7	1/4	-	-	nematodes only	*nhr-84*, nuclear hormone receptor
K06B4.12	1/4	-	-	nematodes only	*twk-34*, tandem pore domain K+ channel
C06E7.6	1/4	-	-	nematodes only	*spe-27*, required for hermaphrodite sperm activation
F45C12.11	1/4	-	-	nematodes only	*sdz-18*, skn-1 dependent zygotic transcript, uncharacterised protein contains BTB/POZ domain
K02F6.3	1/4	-	decreased	nematodes only	WSN domain
C55A1.6	1/4	-	-	nematodes only	uncharacterised protein
T04D1.2	1/4	-	-	nematodes only	protein containing an F-box
Y53F4B.r	1/4	-	-	nematodes only	Y53F4B.14, uncharacterised protein
F41E6.8	1/4	-	-	nematodes only	uncharacterised protein
C25E10.8	1/4	-	-	nematodes only	uncharacterised protein
F36G9.13	1/4	-	-	nematodes only	WSN domain
LLC1.2	1/4	-	-	nematodes only	uncharacterised protein
Y73F8A.r	1/4	-	-	nematodes only	targets ZK1248.7
C35D6.3	1/4	-	-	nematodes only	
F27C8.5	1/4	-	-	nematodes only	
C28D4.5	1/4	-	-	nematodes only	uncharacterised protein
F01G10.5	1/4	-	-	nematodes only	
Y65A5A.3	1/4	-	-	nematodes only	
F52E1.5	1/4	-	-	nematodes only	
K06B4.3	1/4	-	-	nematodes only	
C33A12.4	1/4	-	-	nematodes only	
C31H1.2	1/4	-	-	nematodes only	
C06E7.4	1/4	-	-	nematodes only	
B0244.7	1/4	-	-	nematodes only	
K06H7.1	1/4	-	-	nematodes only	
ZC395.5	1/4	-	-	nematodes only	
C09F5.1	1/4	-	-	nematodes only	
T21B10.4	1/4	-	-	nematodes only	
Y9C2UA.1	1/4	-	-	nematodes only	

T03D8.5	1/4	-	-	*gcy-22*, guanylyl cyclase
Y53F4C.g	1/4	-	decreased	Y53F4B.26, DNA helicase
B0546.4	1/4	-	-	predicted Yippee-type zinc-binding protein
F42A9.4	1/4	-	-	*cyp-33E3*, cytochrome p450 superfamily
Y119D3_463.d	1/4	-	-	*cnc-6*, caenecin peptides
Y59E9_120.d	1/4	-	-	*thn-7* and *thn-5*, thaumatin family
K03D3.3	1/4	-	-	*srz-21*, serpentine receptor class Z
Y7A9C.3	1/4	-	-	*srz-73,* serpentine receptor class Z
F14E5.1	1/4	-	-	permease of the major facilitator superfamily
F41A4.1	1/4	-	-	*cutl-28*, cuticlin-like
Y73B6A.e	1/4	-	-	21ur-12285 (21U-RNA)
Y105C5A.l	1/4	-	-	targets C08E3.13, C08E3.1, Y105C5A.12, Y105C5A.13
H25K10.2	1/4	-	-	
K03D3.9	1/4	-	-	superseded or retired gene name
C33A12.5	1/4	-	-	transposon
C02F4.5	1/4	-	-	
T19C3.6	1/4	-	-	
F19B10.6	1/4	-	-	
F07H5.4	1/4	-	-	
C35D6.5	1/4	-	-	
Y67D8A_349.a	1/4	-	-	no RNAi details
Y38F2A_9208.a	1/4	-	-	no targets identified
Y67D8A_375.b	1/4	-	-	no targets identified
Y53F4B.v	1/4	-	decreased	no targets identified
F48E3.8	1/4	-	-	
F38E1.11	1/4	-	-	
T23F2.2	1/4	decreased	-	
F41C6.3	1/4	-	-	

Table S4. Overlap between genes that are required for expression of *gcs-1p::gfp* in the *prdx-2* mutant (Table S3) and those identified by published RNAi screens of the same RNAi library for different phenotypes. Table shows overlap of genes required for *gcs-1p::gfp* expression in the *prdx-2* mutant in at least 2 out of 4 trials. Comparisons between our gene set and those identified by other screens (by calculation of the cumulative hypergeometric probabilities of genes being identified in each screen by chance) indicates that there is significant overlap between the genes identified by our screen and (i) genes required for resistance to ionising radiation (p=0.0039) and (ii) genes required for the long lifespan of the *daf-2* mutant (p=0.0008). In contrast the representation of genes identified by our screen amongst those identified by the other screens is likely to have occurred by chance (P>0.1).

Screen	No. of genes	overlapping genes	Representation Factor	Hypergeometric probability (P)
Fat reduction[1]	305	6	1.37	0.27
Fat accumulation[1]	111	3	1.88	0.21
Protein aggregation[2]	186	1	0.37	0.93
Increase in lifespan[3]	95	3	2.2	0.156
Increase in lifespan[4]	29	1	2.4	0.34
Tau aggregation[5]	75	1	0.93	0.66
Ionising radiation sensitivity[6]	45	4	6.18	0.0039 (**)
Reduction of *daf-2* lifespan[7]	106	7	4.6	0.0008 (***)
Hypertonic stress sensitivity[8]	34	1	2.05	0.39
Pgpdh-1 suppression[9]	253	4	1.1	0.49
Osmotic sensitivity[9]	84	1	0.83	0.7
PFT sensitivity[10]	106	3	1.97	0.19

[1]Ashrafi K, Chang FY, Watts JL, Fraser AG, Kamath RS, et al. (2003) Genome-wide RNAi analysis of Caenorhabditis elegans fat regulatory genes. Nature 421: 268-272. [2]Nollen EA, Garcia SM, van Haaften G, Kim S, Chavez A, et al. (2004) Genome-wide RNA interference screen identifies previously undescribed regulators of polyglutamine aggregation. Proc Natl Acad Sci U S A 101: 6403-6408. [3]Hamilton B, Dong Y, Shindo M, Liu W, Odell I, et al. (2005) A systematic RNAi screen for longevity genes in C. elegans. Genes Dev 19: 1544-1555. [4]Hansen M, Hsu AL, Dillin A, Kenyon C (2005) New genes tied to endocrine, metabolic, and dietary regulation of lifespan from a Caenorhabditis elegans genomic RNAi screen. PLoS Genet 1: 119-128. [5]Kraemer BC, Burgess JK, Chen JH, Thomas JH, Schellenberg GD (2006) Molecular pathways that influence human tau-induced pathology in Caenorhabditis elegans. Hum Mol Genet 15: 1483-1496. [6]van Haaften G, Romeijn R, Pothof J, Koole W, Mullenders LH, et al. (2006) Identification of conserved pathways of DNA-damage response and radiation protection by genome-wide RNAi. Curr Biol 16: 1344-1350. [7]Samuelson AV, Carr CE, Ruvkun G (2007) Gene activities that mediate increased life span of C. elegans insulin-like signaling mutants. Genes Dev 21: 2976-2994. [8]Choe KP, Strange K (2008) Genome-wide RNAi screen and in vivo protein aggregation reporters identify degradation of damaged proteins as an essential hypertonic stress response. Am J Physiol Cell Physiol 295: C1488-1498. [9]Rohlfing AK, Miteva Y, Moronetti L, He L, Lamitina T (2011) The Caenorhabditis elegans mucin-like protein OSM-8 negatively regulates osmosensitive physiology via the transmembrane protein PTR-23. PLoS Genet 7: e1001267. [10]Kao CY, Los FC, Huffman DL, Wachi S, Kloft N, et al. (2011) Global functional analyses of cellular responses to pore-forming toxins. PLoS Pathog 7: e1001314.

Table S5. RNAi clones which prevented arsenite-induced intestinal *gcs-1p::gfp* expression in wild-type animals. 53 of the 90 RNAi clones that reduced intestinal *gcs-1p::gfp* expression in *prdx-2* mutant animals under normal growth conditions on each of 4 occasions (Table S3) also prevented an increase in intestinal *gcs-1p::gfp* expression in wild-type (N2 *gcs-1p::gfp*) animals treated for 90 minutes with 1mM arsenite. Clones are listed according to the number of times they prevented an increase in intestinal *gcs-1p::gfp* expression out of the 3 times in which they were screened. Each RNAi clone caused intestinal *gcs-1p::gfp* to remain undetectable levels in at least 50% of the RNAi-treated animals. Gene descriptions were derived from WormBase.

RNAi clone	No of occasions on which clone reduced intestinal *gcs-1p::gfp* expression in arsenite-treated wild-type animals	Gene description
F54C1.3	3/3	*mes-3*, member of polycomb-like chromatin repressive complex
B0025.2	3/3	*csn-2,* COP9 signalosome subunit CSN2
R11A5.1	3/3	*apb-3*, adaptin predicted to be involved in formation of intracellular transport vesicles
C17G10.1	3/3	predicted component of NuA3 histone acetyltransferase complex
C34F11.3	3/3	adenosine monophosphate deaminase
C26C6.1	3/3	*pbrm-1*, human polybromo 1, chromatin remodelling complex
C06A8.1	3/3	orthologous to human MSH HOMEOBOX HOMOLOG 1 (MTHFR) mutated in homocystinuria
T05H10.5	3/3	*ufd-2*, ubiquitin fusion degradation protein-2
F13B10.1	3/3	*tir-1*, toll and interleukin-1 receptor domain containing
C16A3.8	3/3	THO complex (transcription factor/nuclear export factor) subunit
K04G7.12	3/3	targets K04G7.11
T28H11.2	3/3	*srm-1*, serpentine receptor class M
D2096.8	3/3	nucleosome assembly protein NAP-1
C35C5.1	3/3	*sdc-2*, represses transcription of X chromosomes to achieve dosage compensation
F46E10.11	3/3	fibrillins and related proteins containing Ca2+ binding EGF-like domains
F47A4.2	3/3	*dpy-22*, homologous to human transcriptional mediator protein TRAP230, also called mdt-12
F22F7.4	3/3	extracellular protein with conserved cysteines
Y102A5A.1	3/3	*cand-1*, (CAND1 [cullin-associated NEDD8-dissociated protein 1] homolog)
ZK792.3	3/3	*inx-9*, innexin, an integral transmembrane channel protein
C28H8.3	2/3	predicted helicase, DEAD box superfamily
T26A5.2	2/3	
C44B9.2	2/3	
Y111B2C.a	2/3	unfamiliar protein
E04F6.3	2/3	*maoc-1*, peroxisomal multifunctional beta oxidation protein
B0286.4	2/3	*ntl-2*, NOT-like complex component, predicted transcriptional regulator
T26A8.4	2/3	
B0416.5	2/3	permease of the major facilitator superfamily
C45H4.1	2/3	*srbc-16*, serpentine receptor class BC like

RNAi clone	No of occasions on which clone reduced intestinal ***gcs-1p::gfp*** expression in arsenite-treated wild-type animals	Gene description
K04D7.1	2/3	*rack-1*, G protein beta subunit-like protein
M01G12.4	2/3	*srw-111*, serpentine receptor class W
T27B7.1	2/3	*nhr-59*, nuclear hormone receptor
ZK617.1	2/3	*unc-22,* twitchin, a giant intracellular protein with multiple fibronectin- and immunoglobulin-like domains and a single protein kinase domain that is homologous to titin
K05D4.4	1/3	*cyp-33D1,* cytochrome P450 CYP2 subfamily
F08B12.2	1/3	*prx-12*, Predicted E3 ubiquitin ligase involved in peroxisome organization
F21H12.4	1/3	*ptc-2*, membrane-protein patched / PTCH
C29E6.1	1/3	*let-653*, mucin-like protein similar to the highly glycosylated mucins secreted by the apical surface of epithelia
F32B6.3	1/3	U5 snRNP-associated RNA splicing factor
W01B6.9	1/3	*ndc-80,* centromere-associated protein HEC1
D1046.1	1/3	mRNA cleavage factor 1 sybunit
F41H10.7	1/3	*elo-5*, fatty acid elongation
T12D8.5	1/3	
F20H11.3	1/3	*mdh-1*, NAD dependent malate dehydrogenase
R12B2.1	1/3	*sma-4*, TGFbeta receptor signalling protein
R148.2	1/3	
K02F3.11	1/3	*rnp-5*, splicing factor
F58B6.1	1/3	
R134.2	1/3	*gcy-2*, guanylyl cyclase
T18H9.1	1/3	*grd-6*, GROUND domains (extracellular cysteine-containing domain sometimes associated with HOG domains)
F25B3.4	1/3	
R13H4.7	1/3	
F57F5.5	1/3	*pkc-1*, protein kinase C
C50E3.8	1/3	
F21F8.5	1/3	

Table S6. Primer sequences used in quantitative pcr reactions.

primer	sequence
act-1 Fwd	5’-TACCCAATTGAGCACGGTAT-3’
act-1 Rev	5’-TTAGCCTTTGGATTGAGTGG-3’
gcs-1 Fwd	5’-AATCGATTCCTTTGGAGACC-3’
gcs-1 Rev	5’-ATGTTGCCCTCGACAATGTT-3’
gst-7 Fwd	5’-GACAGCTTCCACTCCTTGAA-3’
gst-7 Rev	5’-GCACACTTTCCATTGATTCC-3’
dhs-8 Fwd	5’-CTGGAAGTGGAGTCAGAACT-3’
dhs-8 Rev	5’-CTGTAGTTGCTGCTCCTTGA-3’
sdz-8 Fwd	5’-CTCTTGCTGTTGACTTGAAGG-3’
sdz-8 Rev	5’-TTGTTCAACCGTGAGTGCTG-3’
gst-4 Fwd	5’-AACGACTCCATTTGGCCAGC-3’
gst-4 Rev	5’-CAGTTTTTCCAGCGAGTCCA-3’
