# Supplementary material for: Genome-wide screening identifies new genes required for stress-induced phase 2 detoxification gene expression in animals
Source: BMC Biol. 2014 Aug 14;12:64. doi: 10.1186/s12915-014-0064-6 (PMC4160539; doi:10.1186/s12915-014-0064-6)

Figure S1 Functional analysis of genes for which RNAi produced a synthetic lethal phenotype with *prdx-2* (Table S1)

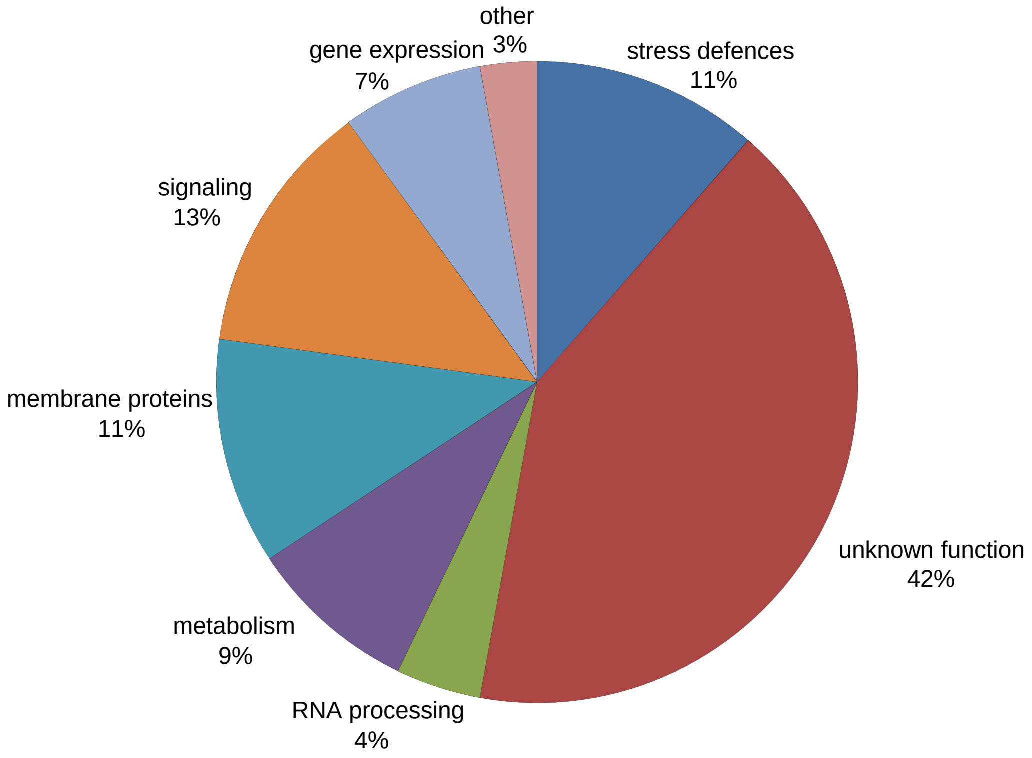

Figure S2. Genes required for intestinal *gcs-1p::gfp* expression in *prdx-2* mutants represent a broad range of functional groups. Pie chart depicting how the genes targeted by the 355 RNAi clones that ablated the increased intestinal gcs-1p::gfp expression in the prdx-2 mutant (Table S3) are distributed between different functional groups. Genes were manually assigned to particular functional groups using GO terms, phenotypic analysis and homology information provided by WormBase (http://www.wormbase.org).

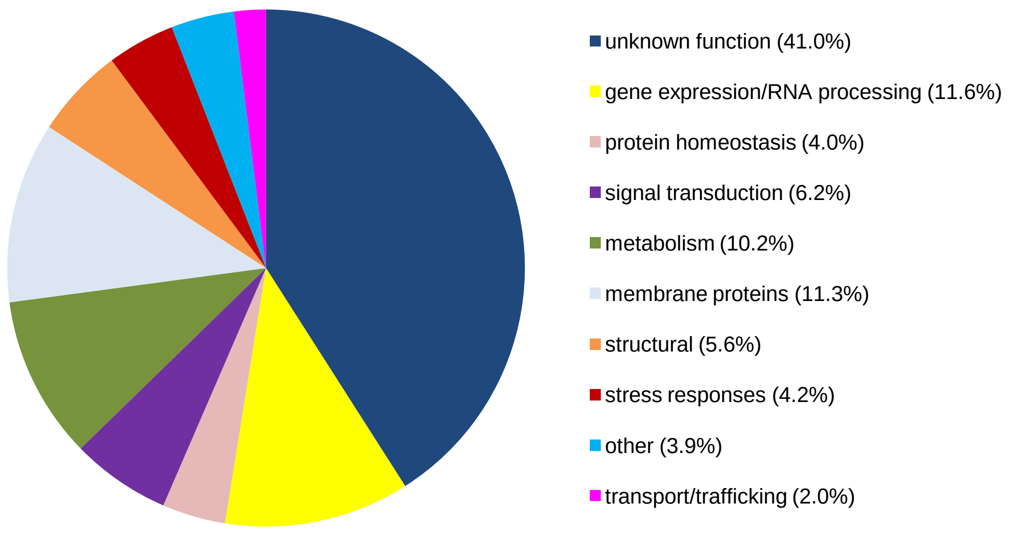

Figure S3 The role of candidate genes identified by screen in the regulation of *gst-4p::gfp* expression in *prdx-2* mutant animals. The effect of selected RNAi clones, targeting 25 candidate regulators (Table 1 and Table S3), on intestinal GFP levels in *prdx-2* (*gk169*) mutants carrying the *gst-4p*::*gfp* reporter gene. **[A]** There is substantially more intestinal expression of *gst-4p::gfp* in *prdx-2* mutant than wild-type worms (compare wt (N2*) gst-4p::gfp and* *prdx-2* (*gk169*) *gst-4p::gfp* maintained on vector control). However, 8 out of 14 candidates identified in each of 4 repeat screens (Table S3; upper panel) and 6 out of 10 less robust candidates, identified in only1 of 4 screens (Table S3; lower panel) significantly reduced the levels of intestinal GFP in *prdx-2* (*gk169*) animals compared with vector control *indicates $p \leq 0.05$, ** indicates $p \leq 0.01$ and *** indicates $p \leq 0.001$(chi^2 test). Each graph represents data acquired in 3 separate experiments. **[B]** Representative images of GFP expression in L3 larval stage wild type (N2) and *prdx-2* (*gk169*) mutant animals containing the *gst-4p*::*gfp* reporter gene, illustrate that; (i) *gst-4p*::*gfp* is expressed at significant levels in the hypodermis and at low levels in the intestine of wild type (N2) worms, that (ii) loss of PRDX-2 dramatically increases intestinal *gst-4p*::*gfp* expression but (iii) that this was abolished by treatment with *cand-1* RNAi. Images were obtained under 10x objective lens using Zeiss Axioskop fluorescent microscope.

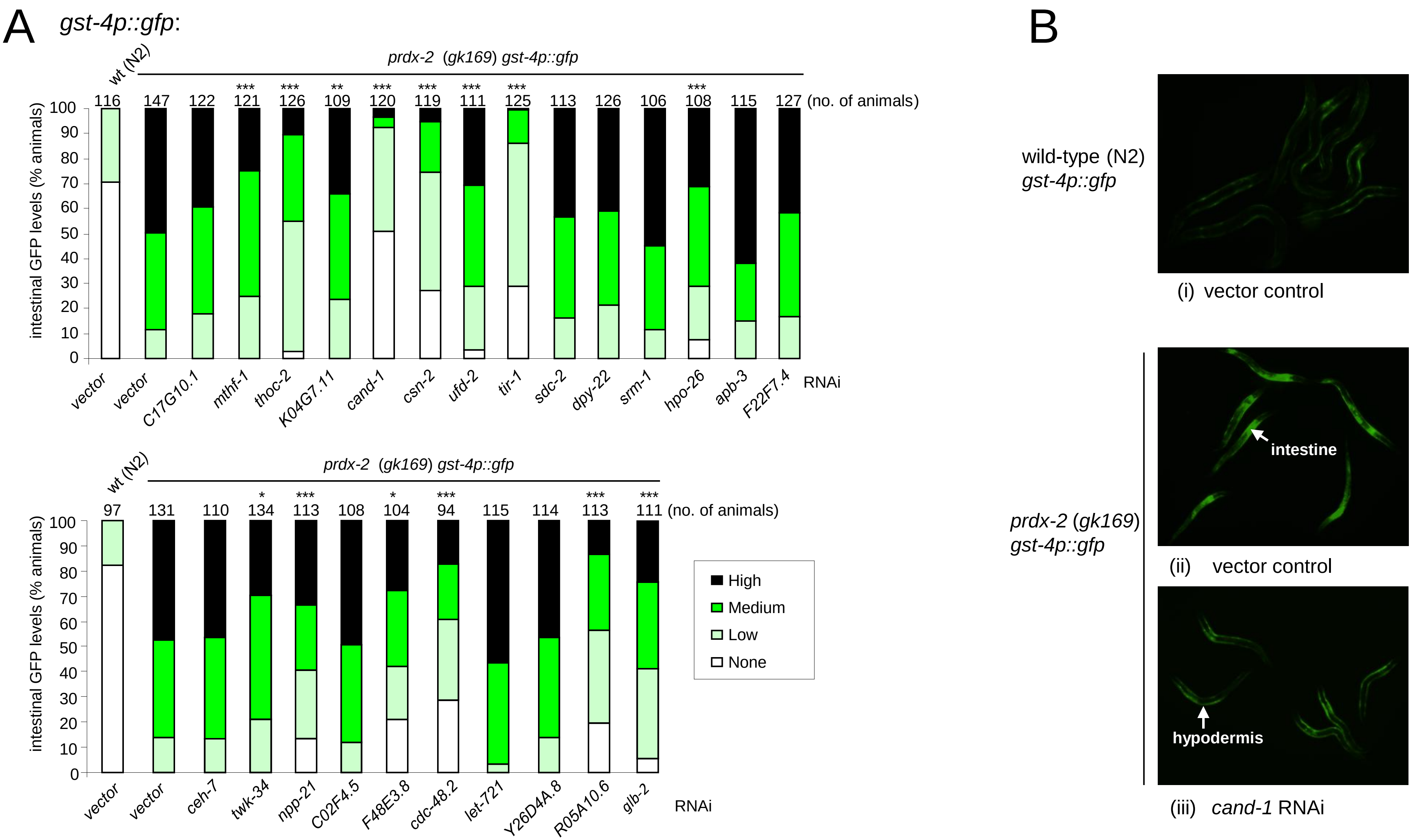

Figure S4 The effect of selected candidate RNAi (Table 1 and Fig. 2B-C) on the arsenite resistance of wild-type animals. Monitoring the survival of wild-type (N2) animals treated with the indicated RNAi or vector control on plates containing 7.5mM sodium arsenite revealed that only a subset of the genes required for arsenite-induced expression of *gcs-1* (Table 1, Fig. 2B) were also required for arsenite tolerance. Each group contained 30-40 animals. Data from 2 representative experiments are shown (for clarity, data collected in a single experiment is shown over 2 separate graphs in [A]) but experiments were repeated multiple times. To account for variations in the rate at which control (vector) animals died in different experiments, % changes in mean survival time compared with control (vector) were calculated for each experiment and mean values determined (see Fig. 2C).

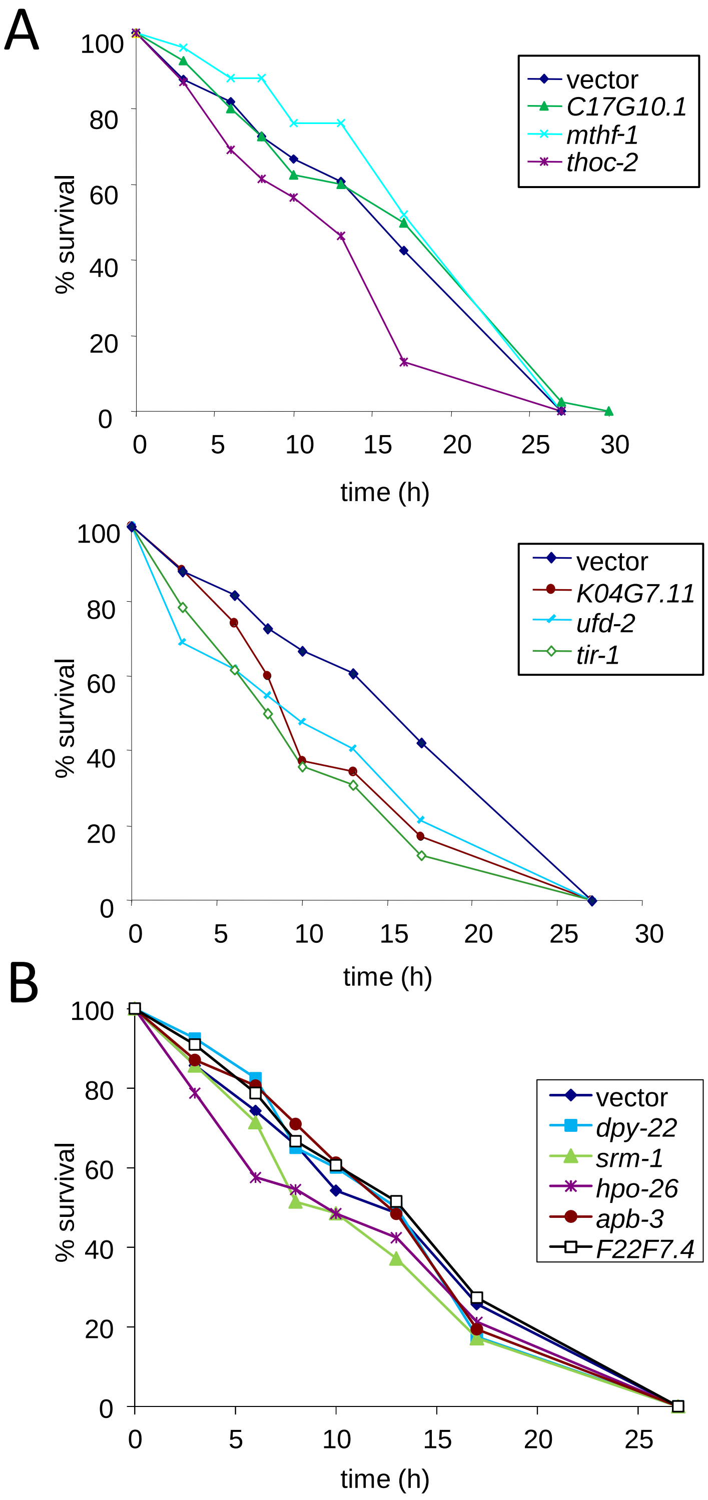

Figure S5. Effect of *csn-2, csn-4* and *csn-5* RNAi on intestinal expression of *gst-4p::gfp* in wild-type animals under normal growth conditions. Results of statistical analysis of data are shown * p<0.05, ** p<0.01 and ***p<0.001 and below (chi^2 test).

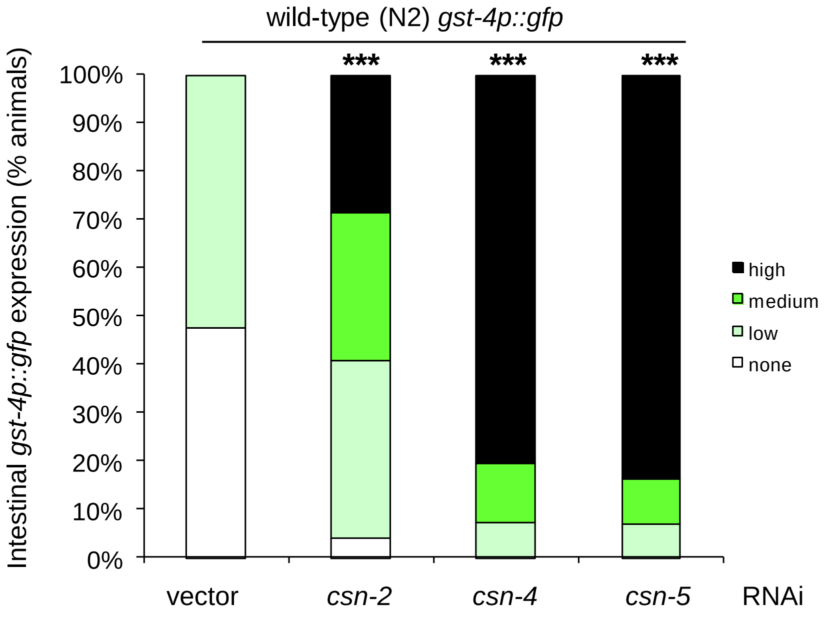

vector vs *csn-2* RNAi - p<0.001
vector vs *csn-4* RNAi - p<0.001
vector vs *csn-5* RNAi - p<0.001

Figure S6 UFD-2 is not required for arsenite-induced PMK-1 phosphorylation. Western blot analysis revealing that basal levels of phosphorylated PMK-1 in wild type (N2) and *ufd-2* (*tm1380*) mutant worms are similar and that the increase in phospho-PMK-1 induced by 5min exposure to 5mM arsenite (As^{3+}) is not inhibited by loss of UFD-2 in *ufd-2* (*tm1380*) mutant animals. The levels of PMK-1 phosphorylation, normalised to tubulin levels, are indicated beneath each lane, relative to those in the wild-type untreated animals.

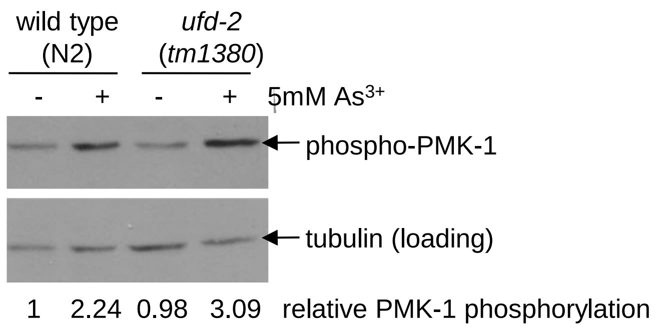

Supplement: Additional file 2: Figure S1. — Functional analysis of genes for which RNAi produced a synthetic lethal phenotype with prdx-2 (Additional file 1: Table S1). Figure S2. Genes required for intestinal gcs-1p::gfp expression in prdx-2 mutants represent a broad range of functional groups. Figure S3. The role of candidate genes identified by the screen in the regulation of gst-4p::gfp expression in prdx-2 mutant animals. Figure S4. The effect of selected candidate RNAi (Table 1 and Figure 2B,C) on the arsenite resistance of wild-type animals. Figure S5. Effect of csn-2, csn-4 and csn-5 RNAi on intestinal expression of gst-4p::gfp in wild-type animals under normal growth conditions. Figure S6. UFD-2 is not required for arsenite-induced PMK-1 phosphorylation. [file 12915_2014_64_MOESM2_ESM.pdf]
